# Structural properties of Au/Cu_2_O catalysts for electrochemical CO_2_ reduction to C_2_ products

**DOI:** 10.1039/d5cy00476d

**Published:** 2025-11-13

**Authors:** Bianca Ligt, Floriane A. Rollier, Tim Wissink, Wei Chen, Jason M. J. J. Heinrichs, Jérôme F. M. Simons, Marta Costa Figueiredo, Emiel J. M. Hensen

**Affiliations:** a Department of Chemical Engineering and Chemistry, Eindhoven University of Technology PO Box 513 Eindhoven 5600 MB The Netherlands e.j.m.hensen@tue.nl; b Eindhoven Institute of Renewable Energy Systems (EIRES), Eindhoven University of Technology PO Box 513 Eindhoven 5600 MB The Netherlands

## Abstract

Improving the selectivity towards multi-carbon products for the electrochemical reduction reaction of CO_2_ (CO_2_RR) with Cu-based catalysts remains a significant topic of scientific interest. It is known that using a secondary metal can provide some control over selectivity, with the structure of the bimetallic catalysts playing an important role in product distribution. In this study, we synthesized Au/Cu_2_O catalysts *via* a precipitation method followed by galvanic replacement using varying Au concentrations. This approach enabled a systematic investigation of the restructuring of Cu_2_O phases decorated with highly dispersed Au, Au–Cu alloys, and Au clusters and their impact on the catalytic activity. Among the tested catalysts, the Cu_2_O catalyst with highly dispersed Au exhibited the highest Faradaic efficiency towards ethylene and ethanol. *In situ* X-ray absorption spectroscopy (XAS) and quasi-*in situ* X-ray photoelectron spectroscopy (XPS) measurements revealed that the presence of Au influenced the reduction of Cu_2_O, where the catalyst with highly dispersed Au displayed the highest fraction of cationic Cu species. Furthermore, *in situ* X-ray diffraction (XRD) was employed to study the structural evolution of crystalline phases of the catalysts during CO_2_RR, which suggests that significant restructuring and redispersion of Au takes place. This work highlights the relevance of *in situ* studies to understand the dynamic interplay between the structure and the catalytic behavior during the reaction.

## Abstract 2

Structure–activity relationships for CO_2_ electroreduction with Cu_2_O catalysts with highly dispersed Au, Au–Cu alloys and Au clusters were investigated using advanced *in situ* characterization techniques.

## Graphical abstract

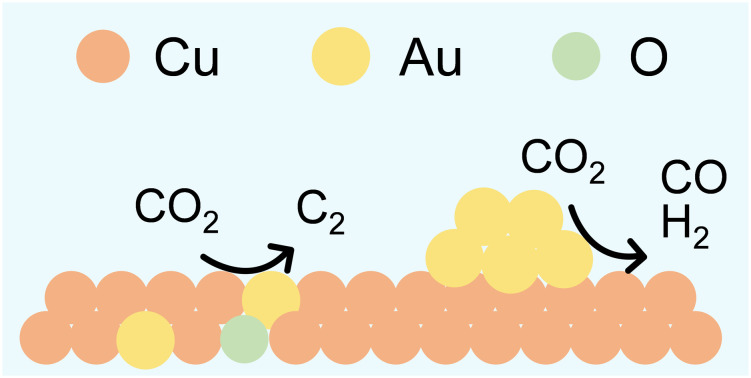

## Introduction

The electrochemical CO_2_ reduction reaction (CO_2_RR) offers a promising method to close carbon cycles.^1^ This process involves the conversion of waste CO_2_ into valuable chemical building blocks and fuels powered by renewable electricity. Copper stands out as an electrocatalyst for this reaction due to its unique ability to form multi-carbon hydrocarbons and oxygenates (*e.g.*, ethylene and ethanol). However, the exclusive formation of one of such products has not been realized yet.^2^

Bimetallic catalysts have been extensively studied to tune the catalytic performance and improve the C_2_ selectivity.^3,4^ The morphology and composition of these catalysts can be readily modified to control the product distribution of the CO_2_RR.^5–7^ In particular, adding Ag,^8,9^ Au,^10,11^ and Zn^12,13^ as a second metal can facilitate the conversion of CO_2_ to carbon monoxide (CO).^14–18^ The enhanced C_2_ selectivity observed with bimetallic catalysts is generally attributed to the role of CO as a crucial intermediate, facilitating C–C bond formation *via* CO dimerization during the CO_2_RR.^19,20^ This constitutes a tandem reaction in which CO_2_ is reduced to CO on the secondary metal, with CO spilling over to the Cu sites where the C_2_ products can be formed. Besides CO spillover, it has been postulated that adding a second metal alters the electronic properties of Cu, impacting the adsorption energies of key intermediates.^21,22^ For instance, Cu–Au alloys were reported to form more CO than monometallic Au catalysts.^23,24^ Cu–Au alloys can also be formed from Au/Cu_2_O catalysts during CO_2_RR, enhancing C_2_ product formation, especially when small amounts of Au are close to Cu sites.^25^

Aspects such as the importance of structural properties and CO spillover mechanism for enhanced C_2_ product formation have also been mentioned for Cu–Ag alloys.^26,27^ For such alloys, it has been discussed that the stabilization of a Cu_2_O overlayer on Ag improves C–C coupling kinetics.^27^ The promoting effect of residual cationic Cu species, presumably due to incomplete reduction of oxidic Cu precursor during CO_2_RR, has also been reported for other Ag/Cu catalysts.^14^ Numerous studies have investigated the role of cationic Cu species, especially Cu^+^, on the selectivity to products containing C–C bonds. It has been mentioned that active sites containing Cu^+^ exhibit different adsorption energies.^27–30^ The partial preservation of Cu^+^ species was also observed with Zn as a secondary metal, although it did not enhance the catalytic performance in the CO_2_RR.^31^ Ngene *et al.* demonstrated almost complete suppression of C_2+_ product formation with a Cu_*x*_Zn_*y*_O catalyst, despite the presence of cationic Cu species and high faradaic efficiency (FE) towards CO.^32^ Comparatively, the effect of Au on the oxidation state of Cu and catalytic performance in the CO_2_RR remains underexplored. Fundamental understanding of the nature of the active sites, restructuring of the catalysts under reaction conditions and the influence of the oxidation state of Cu on the catalytic performance are of great importance to develop electrocatalysts for practical applications.

In this work, we systematically study the structure–activity relationships of well-defined Cu_2_O nanocubes supporting highly dispersed Au particles, Au–Cu alloys, and Au clusters. *In situ* X-ray diffraction (XRD) using synchrotron radiation demonstrates that the Au/Cu_2_O catalysts severely restructure during the CO_2_RR in neutral electrolyte. *In situ* X-ray absorption spectroscopy (XAS) and quasi-*in situ* X-ray photoelectron spectroscopy (XPS) indicate that the presence and amount of Au influence the oxidation state of Cu species.

## Experimental section

### Catalyst and electrode preparation

The preparation of the Cu_2_O nanocubes was done by a procedure reported in literature.^14^ Briefly, 5 mL of 0.1 M CuCl_2_·2H_2_O (99.0%, Sigma-Aldrich) solution was added to 200 mL ultrapure water (18.2 MΩ cm^−1^, Purelab flex, ELGA Labwater) at room temperature. 15 mL of 0.2 M NaOH (Emsure, Merck) solution was added dropwise to the solution under continuous stirring. The solution colored light blue. After 5 min, 10 mL of 0.1 M l-ascorbic acid (99%, Sigma-Aldrich) was added dropwise to the solution. The solution was aged for 1 h and the color turned into yellow-orange. The solution was centrifuged and washed twice with a 1 : 1 ratio (by volume) of ethanol and water and once with ethanol. The final product was dried in a vacuum oven overnight.

The Au/Cu_2_O catalysts were prepared by galvanic replacement. In detail, 25 mg of Cu_2_O was dispersed in 20 mL ethanol and sonicated for 10 min. The desired amount of a 5 mM HAuCl_4_·3H_2_O was pipetted in a separate vial and filled with MilliQ water to a total volume of 5 mL. The resulting solutions with different concentrations of HAuCl_4_·3H_2_O were added dropwise to the Cu_2_O dispersions. The mixtures were further stirred for 30 min at room temperature. The solution was centrifuged and washed once with water and once with ethanol. The final products included Au/Cu_2_O catalysts with highly dispersed Au (1Au/Cu_2_O), Au–Cu alloys (5Au/Cu_2_O) and Au clusters (10Au/Cu_2_O) and were dried in a vacuum oven overnight.

To prepare the electrodes, a catalyst ink was prepared by dispersing 7.5 mg of catalyst in 975 μL isopropanol (99.9%, Sigma-Aldrich) and 25 μL of a Nafion solution (5 wt%, Alfa Aesar) and sonicated for 20 min. 100 μL of catalyst ink was drop-casted on a polished 1 × 1.5 cm glassy carbon electrode to obtain a catalyst loading of 0.5 mg cm^−2^. The electrodes were dried naturally in air.

### High-angle annular dark-field scanning transmission electron microscopy (HAADF-STEM)

The powder samples were dispersed in absolute ethanol and deposited on TEM grids. The images were taken on a CryoTitan (Thermo Fischer Scientific) with an acceleration voltage of 300 kV.

### X-ray photoelectron spectroscopy (XPS)

The X-ray photoelectron spectra of fresh samples were collected with a K-alpha ultra-high vacuum X-ray photoelectron spectrometer by ThermoFisher Scientific equipped with a monochromatic aluminium anode (Kα = 1486.6 eV, 72 W) X-ray source with a spot size of 400 μm and a 180° double focusing hemispherical analyzer with a 128-channel detector. Quasi-*in situ* X-ray photoelectron spectroscopy measurements were carried out on a SPECS system. An ambient pressure electrochemical cell attached to the system allows for performing CO_2_RR in an argon atmosphere. The catalyst ink was drop-casted on a carbon paper (Sigracet 22BB, Ion Power GmbH) to obtain a loading of 1 mg cm^−2^. This electrode served as the working electrode. A reversible hydrogen electrode (RHE, miniRHE Gaskatel) and a Pt foil were used as reference and counter electrode, respectively. A 0.1 M KHCO_3_ was saturated with CO_2_ (pH = 6.8) prior to the measurement. A linear sweep voltammetry measurement (scan rate 5 mV s^−1^) was performed from +0.6 V *vs.* RHE until −0.9 V *vs.* RHE followed by a chronoamperometry measurement at −0.9 V *vs.* RHE for 1 h. A flow of argon was continuously purged in the cell until the transfer of the sample to vacuum was completed. For analysis, a monochromated Al Kα source was used at 50 W. Both fresh and quasi-*in situ* samples were measured at a pass energy of 50 eV. The charging states of elements of interest were analyzed with core-level lines C 1s, O 1s, Cu 2p, Cu LMM and Au 4f. The spectra were processed using the CasaXPS software. All spectra were aligned to the carbon peak (*E* = 284.8 eV). The fitting parameters related to Cu species were taken from literature.^33^ The composition ratio of Cu to Au was calculated by considering the relative sensitivity factors (RSF) of the metals.

### X-ray absorption spectroscopy (XAS)

*In situ* X-ray absorption spectroscopy (XAS) measurements were performed at the ROCK beamline at SOLEIL French national synchrotron facility. A bending magnet was used as an X-ray source, and a channel-cut Si(111) quick-scanning monochromator was used for energy selection. The intensity of the incoming X-rays was measured using an ionization chamber filled with nitrogen. The measurements were employed at the Cu K-edge and Au L_3_-edge in fluorescence mode using a PIPS detector. A home-built electrochemical cell was used for the *in situ* XAS measurements. The catalyst ink was drop-casted on carbon paper to obtain a loading of 0.5 mg cm^−2^ for measurements at the Cu K-edge and 5.0 mg cm^−2^ for measurements at the Au L_3_-edge. These distinct loadings were selected to optimize the signal-to-noise ratio for each edge while minimizing self-absorption effects. A Pt mesh and RHE were used as counter and reference electrode, respectively. During the electrochemical measurements, the electrode was first reduced with a linear sweep from +0.6 V to −0.9 V *vs.* RHE (scan rate 5 mV s^−1^) followed by a chronoamperometry measurement at −0.9 V *vs.* RHE for 15 min. X-ray absorption near-edge structure (XANES) and extended X-ray absorption fine structure (EXAFS) spectra were normalized in the Athena software. The XANES data were analyzed with Multivariate Curve Resolution – Alternating Least Squares (MCR-ALS) analysis with a MATLAB script.^34^ The number of components was determined by principal component analysis (PCA). For the analysis, non-negative and closure constraints of the concentration of the components were used. EXAFS fitting was performed in the *R*-space with the Artemis software, with fitting ranges of *k* = 3 to 12. The amplitude reduction factor *S*_0_^2^ was determined by fitting the Cu foil, Au foil, and Cu_2_O reference data.

### X-ray diffraction (XRD)

X-ray diffraction (XRD) measurements were performed at ID31 beamline of the ESRF synchrotron. An incident photon energy of 75 keV (0.0165 nm) and Pilatus CdTe 2M dectector were used in a Debye–Scherrer geometry. For the *in situ* measurements, a home-built electrochemical cell was used. Polyether ether ketone (PEEK) windows of 250 μm thickness were used to minimize X-ray absorption. To ensure a sufficient signal-to-noise ratio for XRD analysis, the catalyst ink was drop-casted onto carbon paper with a loading of 5 mg cm^−2^, which served as the working electrode. An RHE and a Pt wire were used as the reference and counter electrode, respectively. The electrolyte (0.1 M KHCO_3_) was continuously bubbled with CO_2_ and flown through the cell during the experiments. At first, one cycle was recorded during a cyclic voltammetry measurement between +0.5 V and −0.5 V *vs.* RHE with a scan rate of 2 mV s^−1^. Subsequently, a linear sweep voltammetry measurement was performed from +0.1 V until −1.0 V *vs.* RHE with a scan rate of 2 mV s^−1^. Finally, a chronoamperometry measurement was executed at −0.9 V *vs.* RHE for 10 minutes. A background subtraction was applied to the diffractograms to account for the contributions of the cell, carbon paper and electrolyte.

### Electrochemical measurements

The glassware used for the electrochemical measurements was thoroughly cleaned before the experiments to avoid organic and inorganic contaminations. The organic contaminations were removed by storing the glassware overnight in an aqueous solution of 1 g L^−1^ KMNO_3_ (98%, Alfa Aesar) and 0.5 M H_2_SO_4_ (95–97%, Merck). Subsequently, the solution was drained and residual KMnO_4_ was removed with 10% H_2_O_2_ (33%, VWR). Then, the glassware was boiled three times in ultrapure water.

A Metrohm Autolab PGSTAT302N potentiostat was used for electrochemical CO_2_RR experiments in a gas-tight home-built H-type cell. The cathodic and anodic compartments were separated by an anion exchange membrane (Fumasep FAA-3-PK-130). The membrane was activated in 0.5 M KOH solution for 20 h prior to the measurements. Both compartments were filled with CO_2_-saturated 0.1 M KHCO_3_ solution (pH = 6.8). The volume of the electrolyte in both compartments was 60 mL. A Pt mesh and a Ag/AgCl electrode (3 M KCl, redox.me) were employed as counter and reference electrode, respectively. The working electrode was fixed in a custom-made polyether ether ketone (PEEK) holder with an exposed geometric area of 1 cm^2^. During electrolysis, there was a continuous flow of CO_2_ into the cathodic chamber at a constant rate of 15 mL min^−1^. Before chronoamperometry, the samples were reduced with linear sweep voltammetry (LSV) from 0 V *vs.* Ag/AgCl to the cathodic potential with a scan rate of 5 mV s^−1^. The chronoamperometry measurement was performed at the cathodic potential for 1 h. The measured potential values were converted to RHE values and *iR*-corrected according to the following equation:*E* (V *vs.* RHE) = *E* (V *vs.* Ag/AgCl) + 0.21 V + 0.059 × pH − 0.85 × *iR*The value for *iR* was determined by electrochemical impedance spectroscopy with a frequency range of 1 Hz to 10 kHz. For each potential, three measurements were performed with freshly prepared electrodes under identical experimental conditions to calculate the error bars. The electrochemical surface area (ECSA) was evaluated by measuring the double-layer capacitance in a non-faradaic potential range with LSV at scan rates of 25, 50, 75, 100 and 150 mV s^−1^ after the electrochemical reaction in CO_2_-saturated 0.1 M KHCO_3_ as supporting electrolyte.

### Product analysis

Online gas chromatography (GC), equipped with a thermal conductivity detector (TCD) and a flame ionization detector (FID) with a methanizer, was used for gas product quantification. A gas sample was injected every ∼10 min for analysis. Liquid product analysis was performed with ^1^H-nuclear magnetic resonance spectroscopy (^1^H-NMR) using water suppression mode on a Bruker 400 MHz instrument. 450 μL of electrolyte was mixed with 50 μL of 10 mM dimethyl sulfoxide (DMSO) (>99.9%, Biosolve) and 50 μM phenol (>99%, Sigma-Aldrich) in D_2_O (99.9%, Sigma-Aldrich) as internal standards. The faradaic efficiency (FE) for gas and liquid products was calculated by the charge consumed for the product divided by the total charge:FE = *z* × *n* × *F*/*Q*where *z* represents the number of electrons involved in the formation of the product (*e.g. z* = 2 for CO and *z* = 12 for C_2_H_4_), n is the number of moles for a specific product, *F* is the Faraday constant (96 485 C mol^−1^) and *Q* is the total charge (*C*) for the measurement.

## Results and discussion

The Cu_2_O nanocrystals with cubic shape were prepared by a ligand-free precipitation method.^14^ The Au/Cu_2_O composites were synthesized by a galvanic replacement reaction between HAuCl_4_ and the Cu_2_O nanocrystals (6 H^+^(aq) + 2 AuCl_4_^−^(aq) + 3 Cu_2_O(s) = 6 Cu^2+^(aq) + 8 Cl^−^(aq) + 2 Au(s) + 3H_2_O(l)).^35^ In parallel, the disproportionation reaction of Cu_2_O into Cu and CuO takes place (Cu_2_O(s) = Cu(s) + CuO(s)) in the acidic environment. The galvanic replacement was performed in an ethanol–water mixture, because ethanol is known to slow the reactions, thereby stabilizing the Cu_2_O surface.^36–38^ As a result, the Au and Cu atoms can form Au–Cu alloys at ambient conditions. As the Au content increases, the Au atoms cluster not only with Cu atoms, but also with each other, forming Au particles. For this study, we synthesized Au/Cu_2_O composites with Au contents of 1, 5 and 10 mol% based on the metal content. This resulted in the formation of well-defined Cu_2_O catalysts decorated with highly dispersed Au in 1Au/Cu_2_O, Au–Cu alloys in 5Au/Cu_2_O and Au clusters in 10Au/Cu_2_O, allowing for a systematic study of monitoring the restructuring of these phases during CO_2_RR and its impact on the catalytic performance. The characterization of the as-prepared catalysts will be discussed below.

**Fig. 1:**
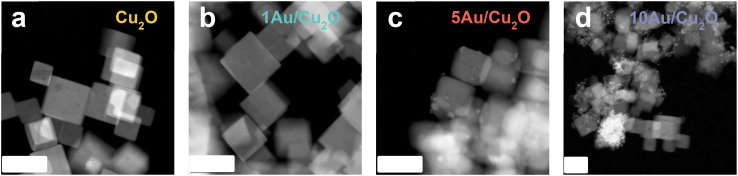
HAADF-STEM images of (a) Cu_2_O, (b) 1Au/Cu_2_O, (c) 5Au/Cu_2_O and (d) 10Au/Cu_2_O. The scale bar corresponds to 50 nm.

The HAADF-STEM images of the freshly prepared catalysts are shown in Fig. 1. Representative bright-field TEM images are shown in Fig. S1. The Cu_2_O nanocrystals display a cubic morphology and are enclosed with six (100) crystal planes.^39^ The cubes have an edge length of approximately 30 nm. Huang *et al.* showed that, although the cubic shape was preserved in the presence of Au, the Cu_2_O surface became rougher.^37^ For 1Au/Cu_2_O, we observed that the Cu_2_O surface looks similar to that of the Au-free sample, likely due to the low Au loading. This suggests a high dispersion of Au atoms on the Cu_2_O nanocubes. At higher Au loading (5 mol% Au), small Au nanoparticles with a size of 5 nm appeared on the Cu_2_O surface. Many such nanometer-sized particles were observed on the sample containing 10 mol% Au. This latter sample also contained some larger agglomerates of Au particles.

**Fig. 2:**
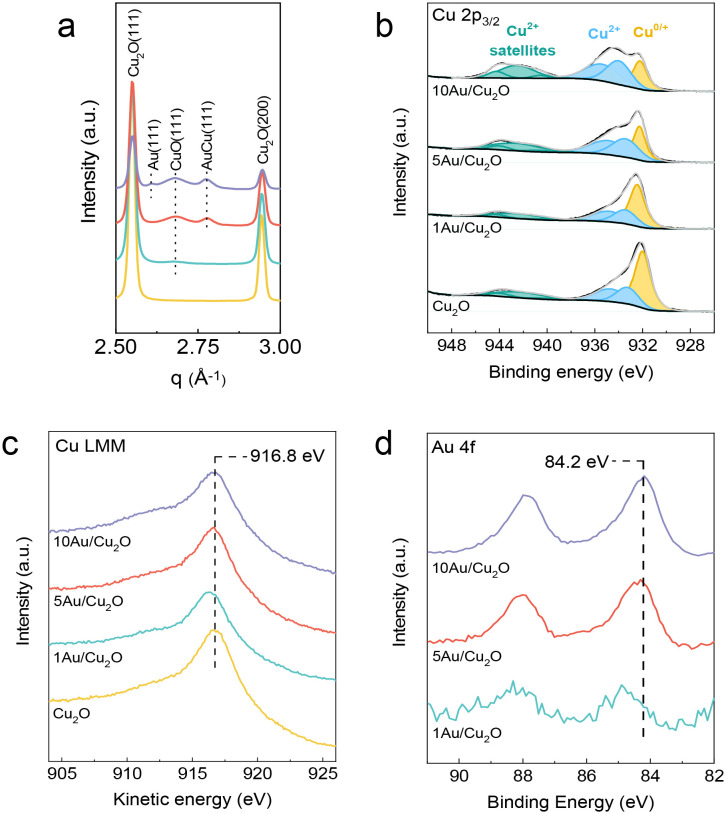
(a) Synchrotron X-ray diffractograms of the as-prepared samples (*λ* = 0.0165312 nm). (b) Cu 2p_3/2_, (c) Cu LMM and (d) Au 4f XPS spectra of the as-prepared samples.

X-ray diffraction (XRD) and X-ray photoelectron spectroscopy (XPS) measurements were performed to identify the presence of a Cu_2_O phase with highly dispersed Au species, Au–Cu alloys and larger Au clusters in the as-prepared samples (Fig. 2). The crystalline phases in the as-prepared Cu_2_O nanocubes and Au/Cu_2_O composites were determined by synchrotron powder XRD. The diffractograms shown in Fig. 2a and S2 clearly demonstrate the crystalline nature of the materials. The main Cu_2_O reflections attributed to the (111) and (200) diffraction planes are found at q values of 2.55 Å^−1^ and 2.94 Å^−1^, respectively. As the patterns only contain reflections of Cu_2_O, it can be concluded that crystalline Au- and Au–Cu-containing phases are absent in the 1Au/Cu_2_O sample, in line with the high Au dispersion suggested by the EM images. In contrast, the XRD patterns of 5Au/Cu_2_O and 10Au/Cu_2_O contain characteristic (111) reflections of AuCu alloy at *q* = 2.78 Å^−1^ and CuO at *q* = 2.68 Å^−1^. The 10Au/Cu_2_O sample also contains a diffraction peak at *q* = 2.60 Å^−1^, which can be assigned to the Au(111) reflection. The low intensity of the peaks related to CuO, AuCu, and Au shows that they are minority phases. The broad peaks indicate the small size of the crystalline domains and suggest the high dispersion of these phases. The sizes of the Cu_2_O and Au/Cu_2_O crystallites derived from XRD data were in the range of 25–30 nm and are in good agreement with electron microscopy data (Table S1). XPS analysis was performed on the fresh samples to study the surface of the catalysts (Fig. 2b–d). From the peak at 932.2 eV in the Cu 2p_3/2_ XPS spectra (Fig. 2b), it can be judged that metallic Cu^0^ and/or Cu^+^ species are present on the surface. Furthermore, Cu^2+^ species were detected as can be seen from the shoulder at 934.0 eV and the characteristic satellite feature in the 939–945 eV range.^33^ The existence of Cu^2+^ species is most likely due to the air exposure of the sample. To distinguish between Cu^0^ and Cu^+^, we analyzed the Cu LMM spectrum (Fig. 2c). This spectrum demonstrated the absence of Cu^0^ species and revealed the presence of a mixture of Cu^+^ and Cu^2+^. With increasing Au content, the fraction of Cu^2+^ species on the surface increased. This is revealed by the corresponding Cu 2p_3/2_ spectra, which display a higher relative intensity of the Cu^2+^ to the Cu^0/+^ peaks and the strong satellite feature. Besides air exposure, Cu^2+^ can be formed by the reactions taking place during Au introduction, *i.e.*, galvanic replacement reaction and Cu_2_O disproportionation, as described earlier.^35^ Au 4f XPS spectra provide evidence for Au species on the surface of the Cu_2_O nanocubes (Fig. 2d). The Au content at the surface determined from these XPS spectra was close to the targeted theoretical values (Table S2). However, at higher Au loadings, the amount of Au ending up in the sample is significantly lower than the targeted value. We surmise that this is caused by the simultaneous formation of CuO due to disproportionation. CuO does not participate in the galvanic replacement reaction, *i.e.*, CuO will not react with HAuCl_4_^−^. The shift of the Au 4f_7/2_ peak from 84.7 to 84.2 eV with increasing Au loading revealed the formation Au–Cu alloys and/or Au clusters.^40–42^ Thus, we speculate that the Au or Au–Cu alloy particles are highly dispersed in the 1Au/Cu_2_O sample. These observations are in line with the results from HAADF-STEM and XRD.

**Fig. 3:**
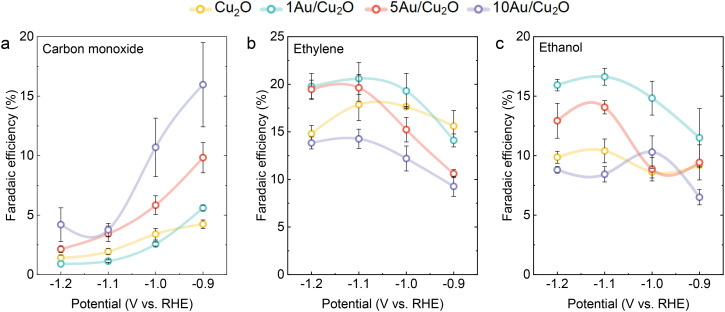
Faradaic efficiencies as a function of the applied potential for (a) carbon monoxide (b) ethylene and (c) ethanol. The potentials were kept constant for 1 hour in CO_2_-saturated 0.1 M KHCO_3_ electrolyte. Solid lines are a guide for the eye.

The performance of the electrocatalysts was assessed using chronoamperometry at fixed potentials in an H-type cell with CO_2_-saturated 0.1 M KHCO_3_ (pH 6.8) as the supporting electrolyte. The catalytic tests were carried out at potentials between −0.9 to −1.2 V *vs.* RHE with intervals of 0.1 V. The FE for CO, ethylene and ethanol obtained after 1 h of CO_2_RR are shown in Fig. 3. The FE for other products formed during electrolysis can be found in Fig. S3. In all cases, C_2_ products were formed during CO_2_RR, as commonly observed for Cu-based catalysts.^43–45^ The introduction of Au and the increase of the Au loading resulted in a higher FE towards CO, especially at less negative potentials. The FE towards ethylene was slightly enhanced for 1Au/Cu_2_O as compared to Cu_2_O. 5Au/Cu_2_O showed only an increase in ethylene formation at more negative potentials. However, the addition of Au to Cu_2_O had a more pronounced effect on the FE towards ethanol. The incorporation of 1 mol% of Au resulted in a significant 1.6-fold increase in ethanol formation at −1.0 and −1.1 V *vs.* RHE, achieving FEs of 15% and 17%, respectively. Notably, a higher Au loading (10 mol% Au) resulted in the lowest FEs for ethylene and ethanol across the entire potential range. To understand the influence of the electrochemical surface area (ECSA) on the product distribution, we carried out double-layer capacitance measurements after CO_2_RR (Fig. S4 and S5 and Table S3). All catalysts exhibit similar ECSAs, suggesting that differences in ECSA cannot account for the observed variations in catalytic performance. Based on the FE data, we hypothesize that the high dispersion of Au or Au–Cu alloys in the as-prepared samples enhances catalytic performance towards C_2_ products. Placing CO-producing sites close to Cu sites facilitates CO spillover, resulting in improved C_2_ FEs. Au–Cu alloys were reported to outperform Au in terms of CO formation, resulting in a high local CO concentration in the double layer, which facilitates C–C coupling.^24^ Furthermore, it is known that the morphology and size of Au particles can affect the CO_2_RR performance, resulting in different CO/H_2_ ratios in the product mixture.^46,47^ We postulate that the larger Au particles in the 10Au/Cu_2_O sample favor CO and H_2_ production, the abundance of such particles on the reduced Cu phase potentially blocking the active Cu sites for C–C coupling. To further examine the interactions between Cu and Au, which could be responsible for the observed differences in the catalytic performance, we employed a variety of *in situ* and quasi-*in situ* characterization methods.

**Fig. 4:**
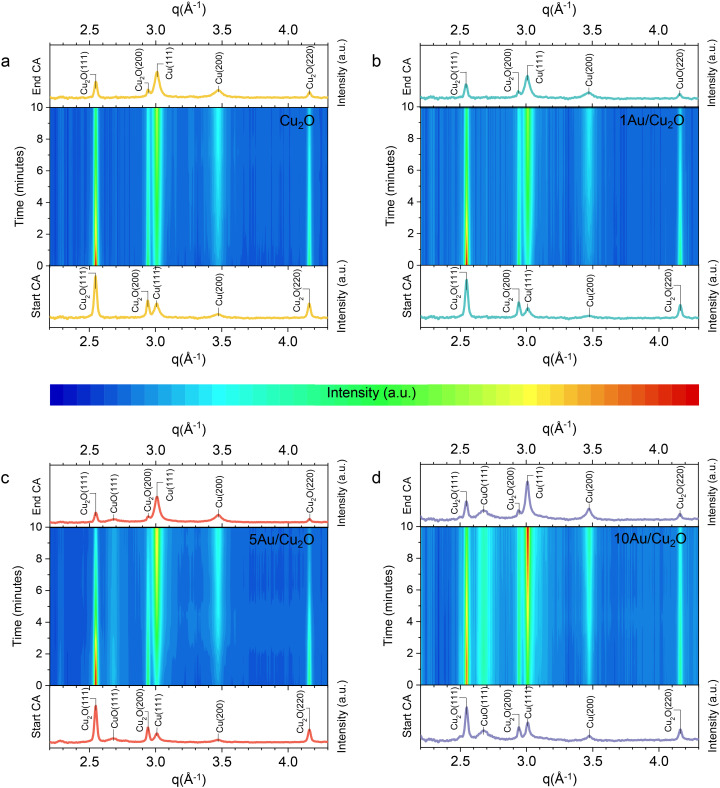
Evolution of the X-ray diffractograms during the chronoamperometry measurement at −0.9 V *vs.* RHE for (a) Cu_2_O, (b) 1Au/Cu_2_O, (c) 5Au/Cu_2_O and (d) 10Au/Cu_2_O.

The evolution of the crystalline phases under CO_2_RR conditions was followed by *in situ* synchrotron XRD. The *in situ* XRD cell has been described in the literature.^48^ For this study, we continuously bubbled CO_2_ in an external reservoir with 0.1 M KHCO_3_ electrolyte (pH 6.8). The diffractograms of the initial state of the catalysts (Fig. S6) are in good agreement with the *ex situ* XRD data shown in Fig. 2a. Due to the relatively low conductivity of the CO_2_-saturated KHCO_3_ electrolyte and the thin electrolyte film in this cell configuration, we recorded a cyclic voltammogram (CV) from −0.5 V to +0.5 V *vs.* RHE at a scan rate of 2 mV s^−1^ (start/stop: +0.1 V *vs.* RHE) as an initial electrochemical assessment. The corresponding diffractograms of the fresh samples and after the CV are shown in Fig. S6. The final recorded diffractogram during the CV shows comparable intensities of the reflections of the Cu or Au–Cu crystalline phases as the fresh sample. A notable exception is the disappearance of the Au(111) reflection in the 10Au/Cu_2_O sample during these measurements, showing that the separate Au particles were not stable during the CV. This may indicate the dissolution of the Au nanoparticles, possibly leading to highly dispersed Au by redeposition. Subsequently, diffractograms were recorded during a linear sweep voltammetry (LSV) measurement from +0.1 V *vs.* RHE to −1.0 V *vs.* RHE with a low scan rate of 2 mV s^−1^, used to follow the bulk reduction during the early stages of CO_2_RR (Fig. S7 and S8). These data show that all samples undergo partial reduction from Cu_2_O to metallic Cu. This follows from the appearance of Cu(111) and Cu(200) reflections at 3.01 Å^−1^ and 3.47 Å^−1^, respectively, and the weakening of the Cu_2_O(111) and Cu_2_O(200) reflections at 2.55 Å^−1^ and 2.94 Å^−1^, respectively. The CuO(111) reflections for 5Au/Cu_2_O and 10Au/Cu_2_O persisted during the LSV. Finally, a chronoamperometric measurement (CA) was conducted at −0.9 V *vs.* RHE for 10 min to simulate the catalytic performance tests. The corresponding time-resolved diffractograms recorded during the CA are shown in Fig. 4 and S9. During the CA, the strong increase of the Cu(111) and Cu(200) reflections shows that Cu_2_O was reduced to metallic Cu. As the diffractograms still contain reflections of Cu_2_O, it can be stated that the reduction of Cu_2_O is not complete. The highest degree of reduction was observed for 10Au/Cu_2_O. Furthermore, the CuO(111) reflection remained visible for the 5Au/Cu_2_O and 10Au/Cu_2_O samples. Besides, the AuCu(111) peak at 2.78 Å^−1^, indicative of a Au–Cu alloy, disappeared during the CA measurement, suggesting that bulk Au–Cu alloys are not stable during CO_2_RR. The crystallite sizes of the various phases in the samples after the CV, LSV and CA measurements were compared to those in the fresh samples (Table S4). The crystallite size derived from the Cu_2_O(111) reflection showed comparable values of *ca.* 20–25 nm before and after the electrochemical measurements. Although the crystallite size derived from the Cu_2_O(200) reflection showed similar values for the Cu_2_O and 1Au/Cu_2_O samples, it increased to roughly 30–35 nm for 5Au/Cu_2_O and 10/Au/Cu_2_O after the CA, hinting at morphological changes during the electrochemical experiments. The size of the metallic Cu particles, determined from the Cu(111) and Cu(200) reflections, showed similar values of approximately 10 nm for all samples after the CA.

**Fig. 5:**
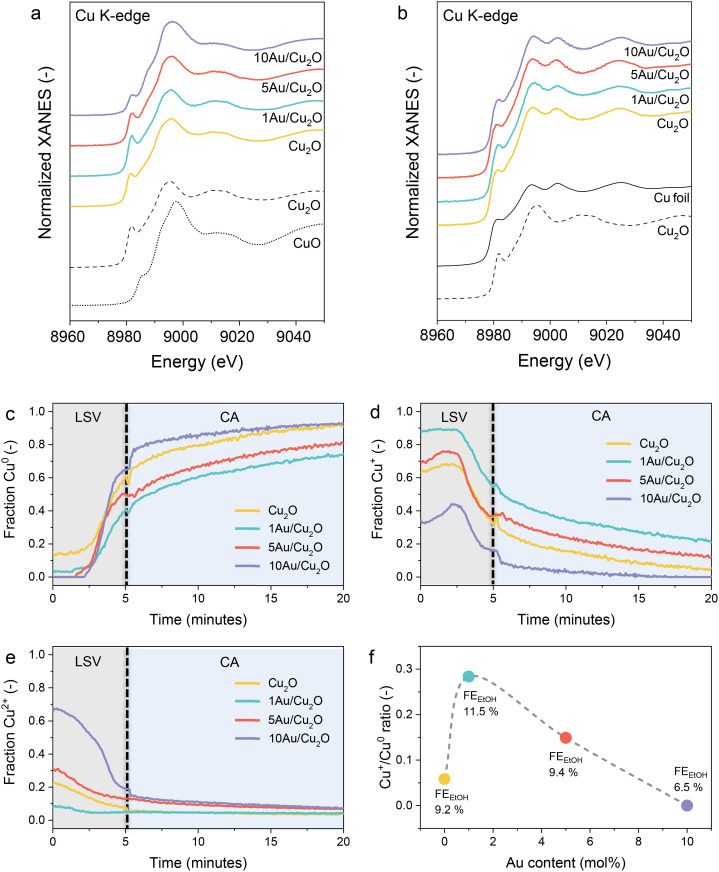
Normalized Cu K-edge XANES spectra of the (a) fresh samples and (b) in the final state during CO_2_RR at −0.9 V *vs.* RHE. References (black) are shown for comparison. Time-dependent fractions of (c) Cu^0^, (d) Cu^+^ and (e) Cu^2+^ during linear sweep voltammetry (LSV) (+0.6 V to −0.9 V *vs.* RHE, scan rate: 5 mVs s^−1^) followed by chronoamperometry (CA) at −0.9 V *vs.* RHE. The dotted lines represent the end of the LSV and the beginning of the CA (f) Cu^+^/Cu^0^ ratio *versus* the theoretical Au loading and the FE for EtOH at −0.9 V *vs.* RHE. Dashed grey line is a guide for the eye.

*In situ* XAS measurements were employed to obtain more detailed information about the chemical state and structure of the catalysts during CO_2_RR. The samples were pre-treated with an LSV measurement from +0.6 V to −0.9 V *vs.* RHE using a scan rate of 5 mV s^−1^, followed by a CA measurement at −0.9 V *vs.* RHE for 15 min in 0.1 M KHCO_3_ under a constant flow of CO_2_. This is the same procedure as used to determine the electrocatalytic performance of the catalysts. The Cu K-edge X-ray absorption near-edge structure (XANES) spectra of the fresh samples and various Cu-containing references are shown in Fig. 5a. The edge energies reveal that Cu is initially present as Cu_2_O, along with some CuO. This phase composition is in good agreement with the XRD and XPS findings (Fig. 2). During the linear sweep from +0.6 V to −0.9 V *vs.* RHE (Fig. S10), the catalysts partially reduce, resulting in a mixture of Cu^2+^, Cu^+^ and Cu^0^ species. Among the samples, the XANES of the 10Au/Cu_2_O sample most closely resembles the Cu foil, indicating a higher degree of reduction compared to the other samples. The final spectra during the CA measurements at −0.9 V *vs.* RHE for all samples are compared to relevant Cu, Cu_2_O, and CuO reference spectra in Fig. 5b. These spectra resemble that of the Cu foil. However, some differences can be observed in the pre-edge feature and the relative intensities of the edge features depending on the sample. The magnitude of these differences was estimated by multivariate curve resolution (MCR) analysis.^34^ The XANES spectra can be described by three components (Fig. S11a), which are in good agreement with the Cu foil, Cu_2_O and CuO references (Fig. S11b). The time-dependent XANES spectra during the LSV and CA measurements are shown in Fig. 5c–e. During the LSV, a mixture of CuO, Cu_2_O and Cu^0^ was formed. The samples further reduced during the CA, as evidenced by the increase in the Cu^0^ fraction at the expense of the Cu^+^ (Cu_2_O) and Cu^2+^ (CuO) fractions. After the electrochemical measurements, the 1Au/Cu_2_O sample contained the highest fraction of Cu^+^. Besides, some Cu^2+^ species also remained in the 5Au/Cu_2_O and 10Au/Cu_2_O samples, which is in line with the observation of residual CuO by *in situ* XRD. Fig. 5f shows the Cu^+^/Cu^0^ ratio as a function of the Au loading. These data show that the Cu^+^/Cu^0^ ratio decreases in the order 1Au/Cu_2_O ≫ 5Au/Cu_2_O > Cu_2_O ≫ 10Au/Cu_2_O. This trend is similar to the order in the FE towards C_2_ products during the CO_2_RR (Fig. 3), which indicates a possible correlation between C–C coupling and the amount of Cu^+^ species. While the beneficial role of Cu^+^ species in enhancing the C_2_ product selectivity is well established^28–30^ such a correlation has not been consistently observed in all studies.^25,32^ For instance, Rettenmaier *et al.* did not report a direct relationship between Cu^+^ species and improved FE for ethylene or ethanol under their experimental conditions.^25^ We speculate that such discrepancies may arise from variations in catalyst preparation methods, reaction conditions or the characterization techniques employed.

**Fig. 6:**
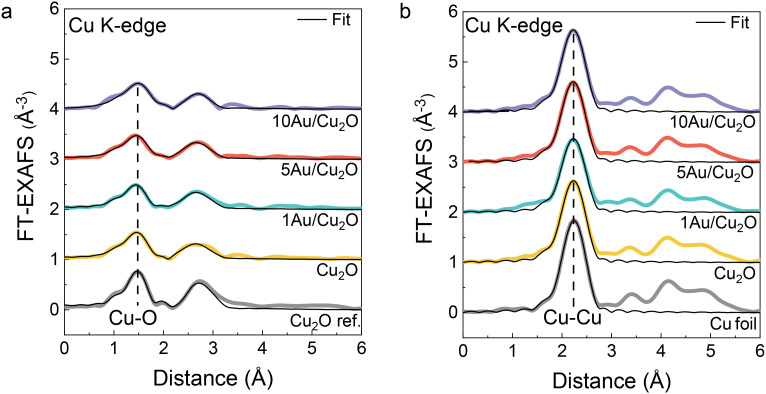
Fourier-transformed Cu K-edge EXAFS of the (a) fresh samples and (b) in the final state during CO_2_RR at −0.9 V *vs.* RHE with the corresponding fits (black). The reference spectra of Cu_2_O and Cu foil are shown for comparison.

Fourier-transformed extended X-ray absorption fine structure (FT-EXAFS) of the Cu_2_O nanocubes and Au/Cu_2_O composites before and after CO_2_RR were analyzed to understand the coordination environment of the Cu species (Fig. 6 and S12). According to the Cu K-edge FT-EXAFS before CO_2_RR, the fresh catalysts are similar to the measured Cu_2_O reference, which agrees with the MCR-ALS XANES analysis. The main contributions at 1.5 Å and 2.8 Å correspond to Cu–O and Cu–Cu bonds, respectively, in Cu_2_O. The EXAFS fitting parameters are presented in Table S5. The Cu–O coordination number (*N*_Cu–O_) of the fresh samples is approximately 2, closely matching that of bulk Cu_2_O. The Cu–O distance (*R*_Cu–O_) was determined to be 1.86 Å, which aligns with the Cu–O distance in bulk Cu_2_O.^49^ During the CA at −0.9 V *vs.* RHE, a new intense contribution emerged at 2.2 Å in the Cu K-edge FT-EXAFS for all samples. This peak can be ascribed to the first Cu–Cu shell in metallic Cu.^50^ The highest Cu–Cu coordination number (*N*_Cu–Cu_) for this metallic Cu shell was observed for 10Au/Cu_2_O (8.7 ± 0.5). Lower *N*_Cu–Cu_ values were reported for Cu_2_O (8.5 ± 0.6), 1Au/Cu_2_O (7.4 ± 0.6) and 5Au/Cu_2_O (8.5 ± 0.6). The lowest *N*_Cu–Cu_ for 1Au/Cu_2_O can be explained by partial preservation of the Cu_2_O phase. The Au L_3_-edge XAS spectra of the Au/Cu_2_O catalysts, recorded before and during CO_2_RR, are shown in Fig. S13. Although the Au signal is relatively weak due to the low Au content, the XANES spectra of the fresh samples exhibit a notable increase in the white-line intensity between 11 920 and 11 940 eV compared to the Au foil reference (Fig. S14a). This increase is indicative of electron transfer from Au to Cu, which is commonly associated with Au–Cu alloy formation.^35,51^ During CO_2_RR, a clear decrease in white-line intensity is observed for 10Au/Cu_2_O (Fig. S14c), suggesting a shift toward more metallic Au under reaction conditions. For 5Au/Cu_2_O (Fig. S14b), this trend is more difficult to resolve, likely due to the lower Au content and associated signal-to-noise limitations. However, the FT-EXAFS data also reveal significant differences in the local structure of Au in the as-prepared samples relative to Au foil, with both spectra only beginning to resemble Au foil under CO_2_RR conditions (Fig. S13d, Table S6). These observations suggest dynamic restructuring and possible dealloying during CO_2_RR, which is in line with the *in situ* XRD results. For the 1Au/Cu_2_O sample, reliable data acquisition during CO_2_RR was challenging due to the low Au content, resulting in poor signal-to-noise ratios. Therefore, this data was excluded from our analysis.

**Fig. 7:**
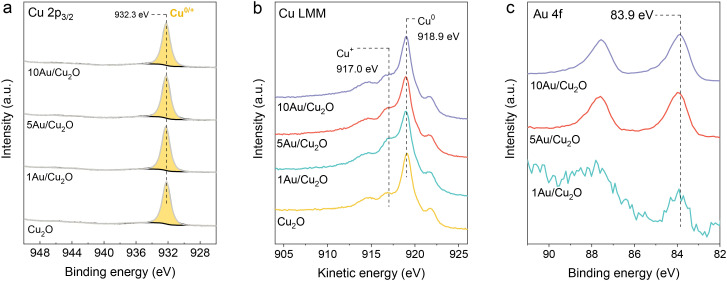
Quasi-*in situ* XPS spectra of the (a) Cu 2p_3/2_, (b) Cu LMM and (c) Au 4f regions (spectra recorded after 1 h CO_2_RR at −0.9 V *vs.* RHE, transfer through vacuum system without air exposure).

The surface composition and the chemical state of the electrocatalysts after 1 h of CO_2_RR at −0.9 V *vs.* RHE were investigated with quasi-*in situ* XPS. A CO_2_-saturated 0.1 M KHCO_3_ solution was used as a supporting electrolyte. The quasi-*in situ* approach allows for the analysis of the surface of the samples without exposure to air. The XPS spectra were recorded directly after the electrochemical measurements and sample transfer to the analysis chamber, performed under an inert atmosphere of Ar to prevent oxidation of the samples. The Cu 2p_3/2_ and Cu LMM spectra in Fig. 7a and b show that all Cu^2+^ species at the surface were reduced, as follows from the absence of the characteristic peaks and satellite feature corresponding to Cu^2+^.^33^ The single Cu 2p_3/2_ feature at 932.3 eV for all catalysts indicates that the surface of the samples only contained Cu^+^ and/or Cu^0^ species. The Cu LMM spectra show that the surface is predominantly made up of metallic Cu (Fig. 7b). The relative intensities of the features at kinetic energies of 917.0 eV and 918.9 eV suggest the presence of residual surface Cu^+^ species. The 1Au/Cu_2_O sample contained the largest amount of Cu^+^ species, which is consistent with the observations from the XAS analysis. The Au 4f spectra shown in Fig. 7c reveal that Au is in the metallic state. The surface composition ratios after CO_2_RR were comparable to those in the fresh samples (Table S2). This indicates that, despite the disappearance of bulk Au according to *in situ* XRD, Au dissolves and redeposits on the surface. All catalysts displayed a Au 4f_7/2_ maximum peak intensity at a binding energy of 83.9 eV. The shift towards lower binding energies for 1Au/Cu_2_O, compared to the fresh sample, can indicate sintering of the dispersed Au particles during CO_2_RR.

### General discussion

This work shows that Au can have a promoting role in Cu-based electrocatalysts for CO_2_RR, where a 1.6-fold increase in the FE towards ethanol was observed for the 1Au/Cu_2_O sample as compared to Cu_2_O alone. Au is introduced on Cu_2_O nanocubes by galvanic replacement, which results in Au and Au–Cu particles dispersed on Cu_2_O. Under CO_2_RR conditions, the combined results from (quasi-)*in situ* spectroscopy reveal that a significant fraction of Cu_2_O is reduced to metallic Cu, while residual Cu^+^ surface species remain and redeposition of Au occurs. Although the experimental protocols and conditions of each technique differ slightly, they offer a complementary and coherent view of the dynamic evolution of the catalysts. This integrated approach provides valuable insights into the much-debated origin of Au's promoting effect on Cu. For instance, it has been suggested that changes in the binding energies of adsorbates and key reaction intermediates at the Cu–Au interface facilitate C–C coupling.^41,52^ This may be linked to the presence of a Au–Cu alloy, which was also observed by XRD. Others pointed out that C–C coupling is faciliated by a high dispersion of the CO-producing metal on Cu, enhancing CO spillover.^16,25^ Au is a selective electrocatalyst for the reduction of CO_2_ to CO, typically exhibiting CO FEs higher than 80% in the potential range of −0.5 V to −0.9 V *vs.* RHE.^11,53,54^ A high local concentration of CO at the surface of the reduced Au/Cu_2_O catalysts promotes C–C coupling at less negative potentials than in the absence of Au.^55^ This effect was demonstrated by a study of Jaramillo's group, who showed that a bimetallic Au/Cu catalyst prepared by chemical vapor deposition of Au on a polycrystalline Cu foil displayed higher FEs towards C_2+_ alcohols at lower overpotentials than the Cu foil without Au.^18^ Our 1Au/Cu_2_O sample exhibited a higher FE towards ethanol at −0.9 V *vs.* RHE compared to the Cu_2_O sample, with the promoting effect of Au becoming more substantial at more negative potentials. Besides CO spillover, it has been frequently put forward that residual cationic Cu^+^ species in the reduced Cu catalysts are associated with higher rates of C–C coupling during CO_2_RR. Our observations from *in situ* XAS and quasi-*in situ* XPS measurements indicate that Au stabilizes residual Cu^+^ species at the Cu surface under reducing conditions. Stabilization of cationic Cu species by Ag under CO_2_RR conditions has previously been reported, also resulting in enhanced C_2_ product formation.^14,26,27^ Density function theory (DFT) calculations have indicated that Cu^+^ can stabilize CO on the Cu surface, thereby promoting its subsequent hydrogenation to *CHO and coupling with *CO, and suppressing the competitive HER reaction.^56–58^ Although it is not possible to distinguish the roles of CO spillover and Cu^+^ species to enhanced C_2_ product formation, our findings show that a too high Au content leads to a smaller promoting effect and a shift of the product distribution from C_2_ products to CO. This is most likely due to the increasing abundance of CO-producing Au particles, which likely cover the Cu sites responsible for further CO reduction reaction, including C–C coupling reactions.

## Conclusion

In this study, we demonstrated the effectiveness of bimetallic Au/Cu_2_O catalysts for the electrochemical reduction of CO_2_. By employing various *ex situ* and (quasi-)*in situ* measurements, we show that galvanic replacement of Au results in the formation of highly dispersed Au, Au–Cu alloys, and Au clusters. Under CO_2_RR conditions, pronounced restructuring of the catalysts is observed, including dealloying of AuCu phases and redispersion of Au. The Cu_2_O sample with highly dispersed Au (1Au/Cu_2_O) showed the highest FE towards the desired C_2_ products, while higher Au loadings (10Au/Cu_2_O) decreased the C_2_ FE, along with an increased FE towards CO and H_2_. Here, highly dispersed Au was proposed to enhance the CO_2_RR performance by providing active sites for the reduction of CO_2_ to CO, which can spill over to the nearby Cu sites. In addition, the highly dispersed Au helps stabilize Cu^+^ species, which were linked to improved C_2_ FE. Further increasing the surface coverage of Au improved CO formation rates at the expense of the number of Cu^+^ species, resulting in poorer C_2_ FE. This work sheds light on the dynamics of the catalytic structure during electrolysis by utilizing a variety of advanced *in situ* characterization techniques. These insights highlight the effectiveness of bimetallic Cu-based catalysts, which will aid in the design of more active and selective catalysts for the CO_2_RR.

## Author contributions

Bianca Ligt (conceptualization, investigation, methodology, data analysis, interpretation, and writing the original draft), Floriane A. Rollier (XRD), Tim Wissink (XAS), Wei Chen (quasi-*in situ* XPS), Jason M. J. J. Heinrichs (HAADF-STEM), Jérôme F. M. Simons (XAS data analysis), Marta Costa Figueiredo (conceptualization, funding acquisition, review & editing), and Emiel J. M. Hensen (conceptualization, funding acquisition, review & editing).

## Conflicts of interest

There are no conflicts to declare.

## Supplementary Material

CY-015-D5CY00476D-s001

## Data Availability

The data supporting this article have been included as part of the supplementary information (SI). Supplementary information is available. See DOI: https://doi.org/10.1039/d5cy00476d.
