# Supplementary material for: Structural properties of Au/Cu2O catalysts for electrochemical CO2 reduction to C2 products
Source: Catal Sci Technol. 2025 Nov 13;15(24):7452–63. doi: 10.1039/d5cy00476d (PMC12612985; doi:10.1039/d5cy00476d)
Supplement: CY-015-D5CY00476D-s001 [file CY-015-D5CY00476D-s001.pdf]

Supplementary Information (SI) for Catalysis Science & Technology.
This journal is © The Royal Society of Chemistry 2025

Electronic Supplementary Information (ESI)

Structural Properties of Au/Cu_2O Catalysts for Electrochemical CO_2 Reduction to C_2 Products

Bianca Ligt, Floriane A. Rollier, Tim Wissink, Wei Chen, Jason M.J.J. Heinrichs, Jérôme F.M. Simons, Marta Costa Figueiredo, Emiel J.M. Hensen*

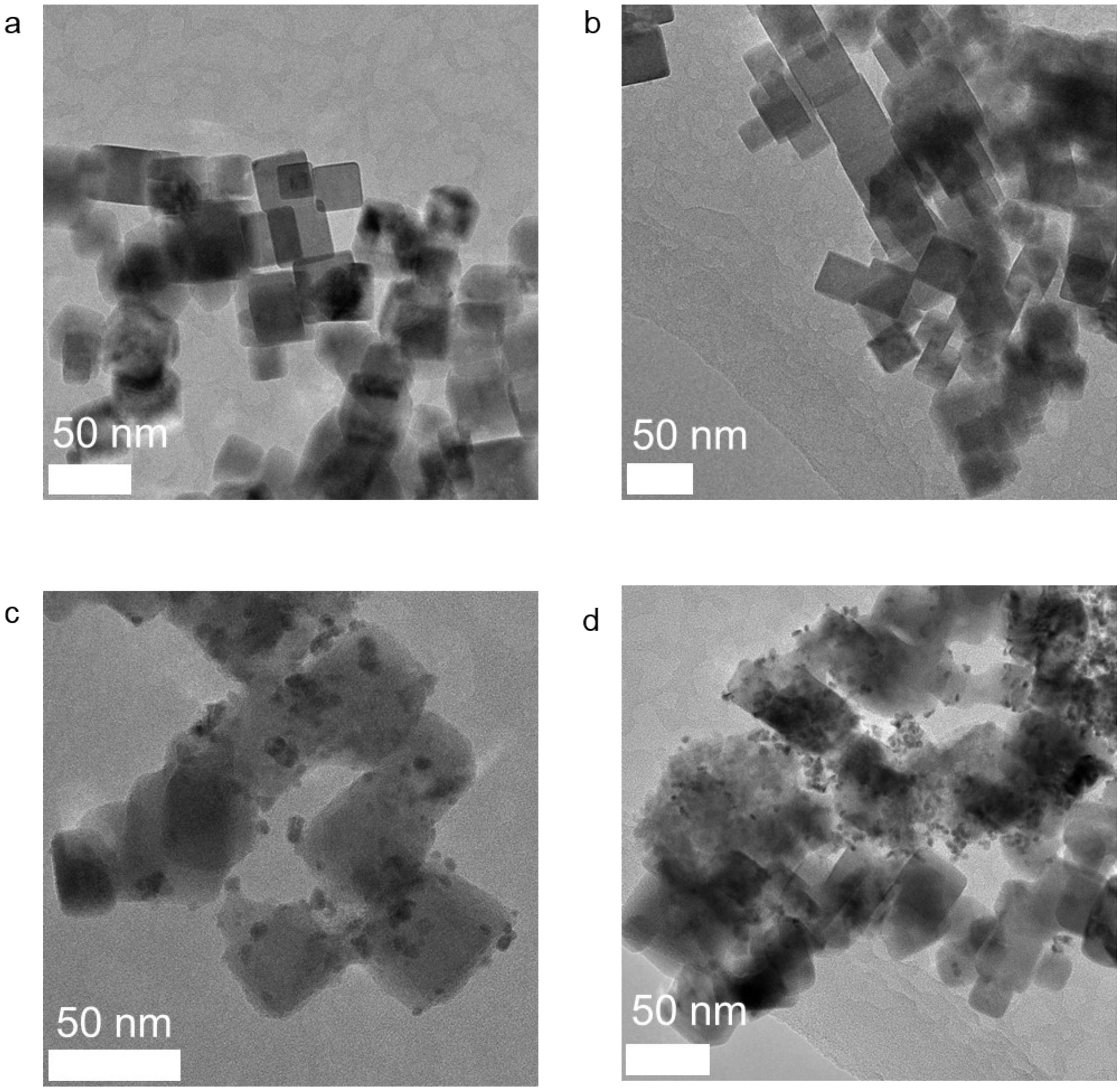

Fig. S1 Bright field-TEM images of (a) Cu_2O, (b) $1Au/Cu_2O$, (c) $5Au/Cu_2O$ and (d) $10Au/Cu_2O$.

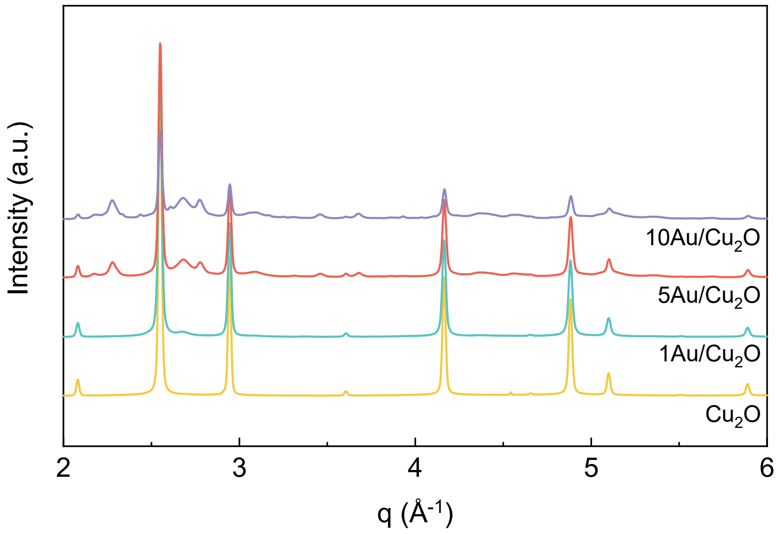

Fig. S2 X-ray diffractograms of the fresh samples recorded in q-range of 2 $Å^{-1}$ to 6 $Å^{-1}$.

Table S1 Crystallite sizes for the fresh samples.

Sample	Cu_2O (111) crystallite size (nm)	Cu_2O (200) crystallite size (nm)	CuO (111) crystallite size (nm)	AuCu (111) crystallite size (nm)
Cu_2O	28.5	29.6	-	-
1Au/Cu_2O	25.0	26.8	-	-
5Au/Cu_2O	25.3	25.7	5.7	13
10Au/Cu_2O	25.4	25.8	5.5	11.6

Table S2 Au at % as determined from the XPS data.

Sample	As-prepared Au at %	After CO_2RR Au at%
1Au/Cu_2O	0.7	0.6
5Au/Cu_2O	4.8	5.2
10Au/Cu_2O	8.1	9.4

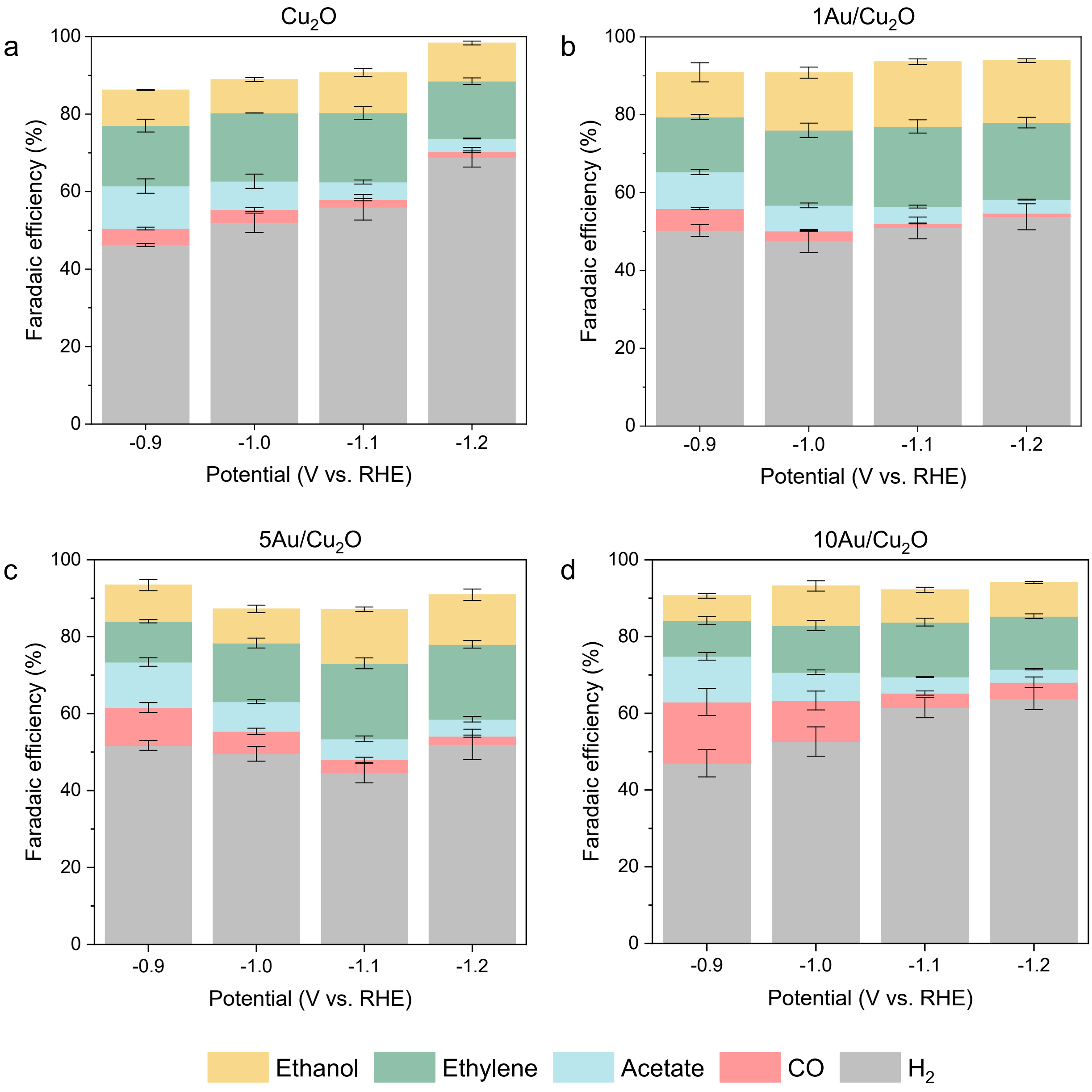

Fig. S3 Faradaic efficiencies for (a) Cu_2O, (b) $1Au/Cu_2O$, (c) $5Au/Cu_2O$ and (d) $10Au/Cu_2O$.

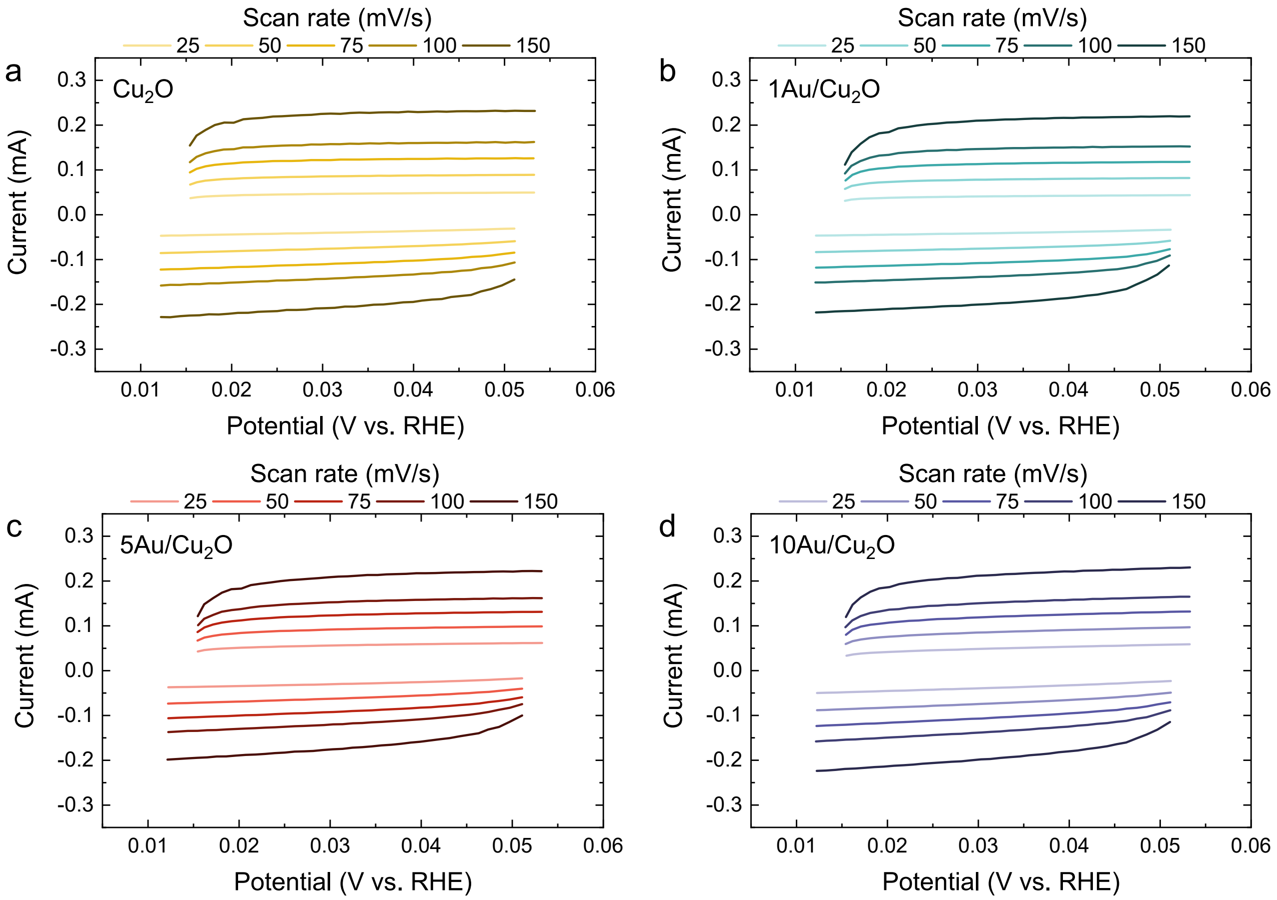

Fig. S4 ECSA measurements of (a) Cu_2O, (b) $1Au/Cu_2O$, (c) $5Au/Cu_2O$ and (d) $10Au/Cu_2O$.

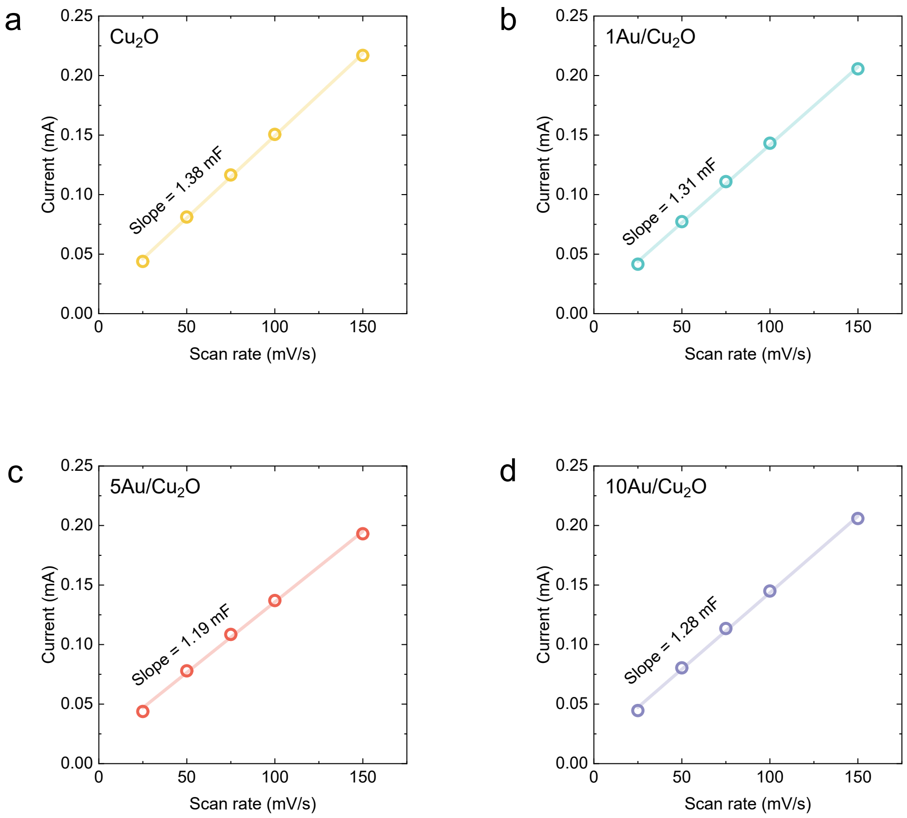

Fig. S5 Slope of the current vs. scan rate for (a) Cu_2O, (b) $1Au/Cu_2O$, (c) $5Au/Cu_2O$ and (d) $10Au/Cu_2O$.

Table S3 Calculated values of the electrochemical active surface area for the samples after CO_2RR. A reference value for the capacitance was taken from literature.[1]

Sample	ECSA (cm^2)	Error (cm^2)
**Cu_2O**	45.3	1.6
**$1Au/Cu_2O$**	45.7	1.3
**$5Au/Cu_2O$**	43.5	2.1
**$10Au/Cu_2O$**	44.5	1.5

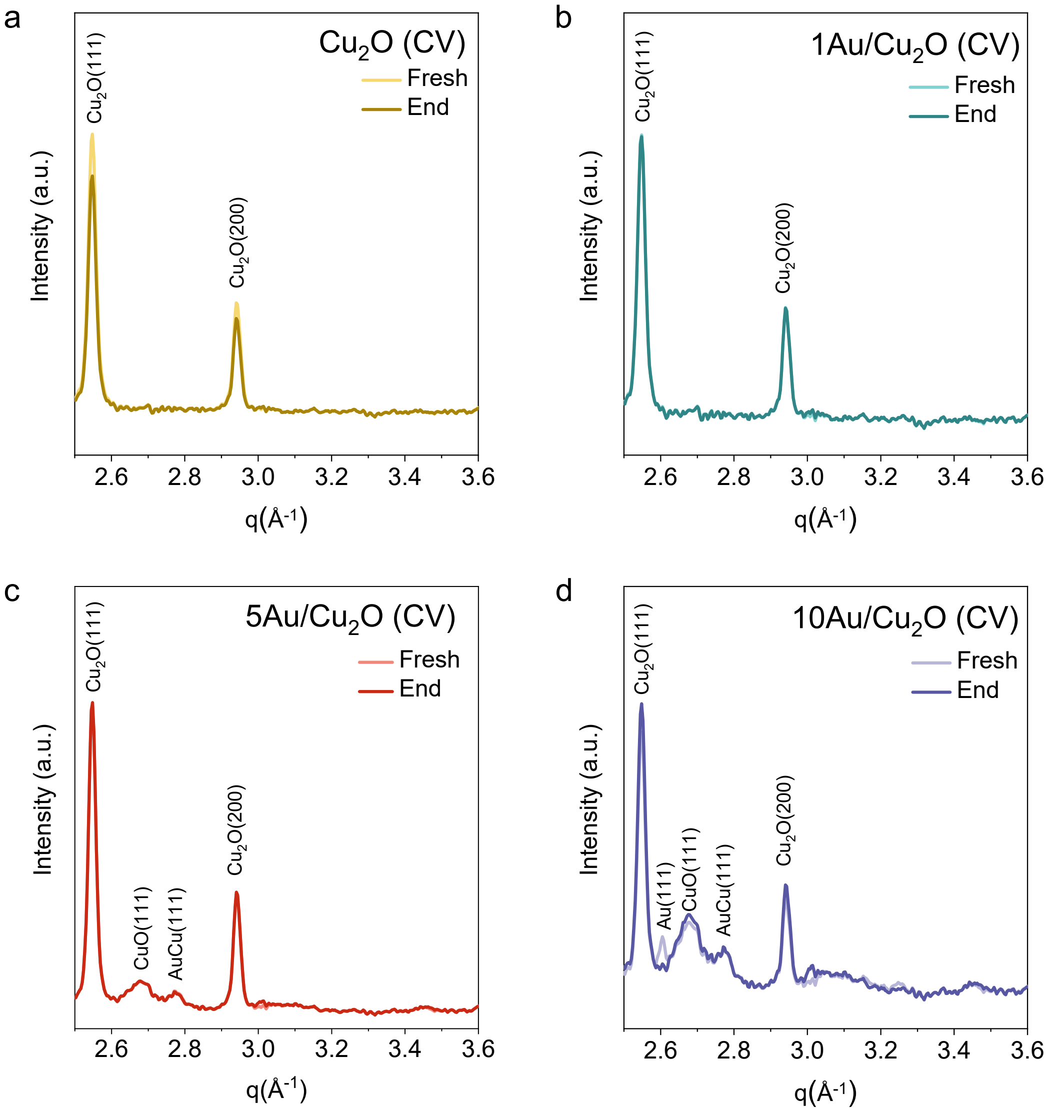

Fig. S6 Recorded diffractograms of the fresh samples and at the end of the CV (-0.5 V to +0.5 V vs. RHE, scan rate: 2 mV/s) for (a) Cu_2O, (b) $1Au/Cu_2O$, (c) $5Au/Cu_2O$ and (d) $10Au/Cu_2O$.

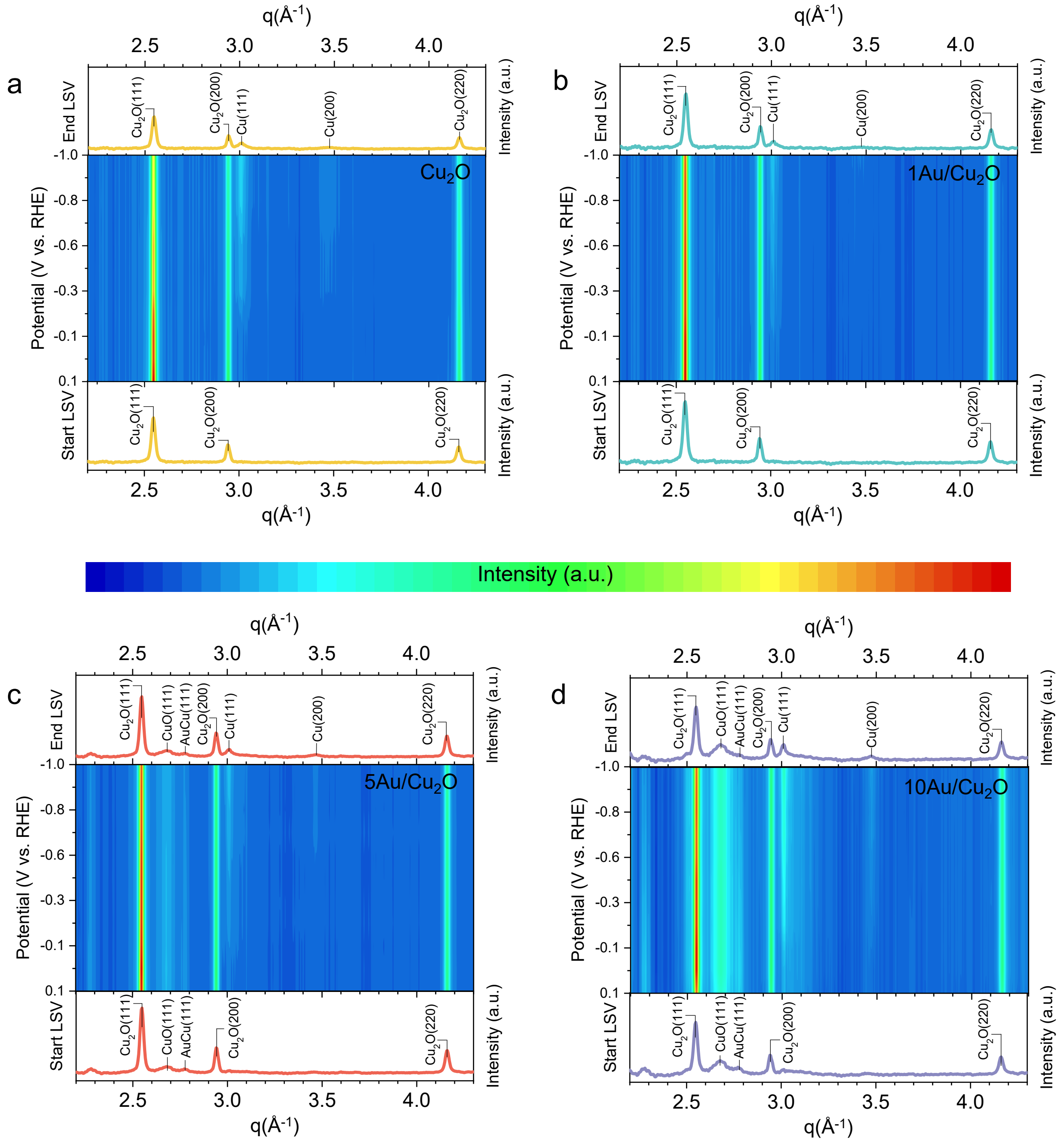

Fig. S7 Evolution of the diffractograms during the LSV from +0.1 V to -1.0 V vs. RHE (2 mV/s) for (a) Cu_2O, (b) $1Au/Cu_2O$, (c) $5Au/Cu_2O$ and (d) $10Au/Cu_2O$.

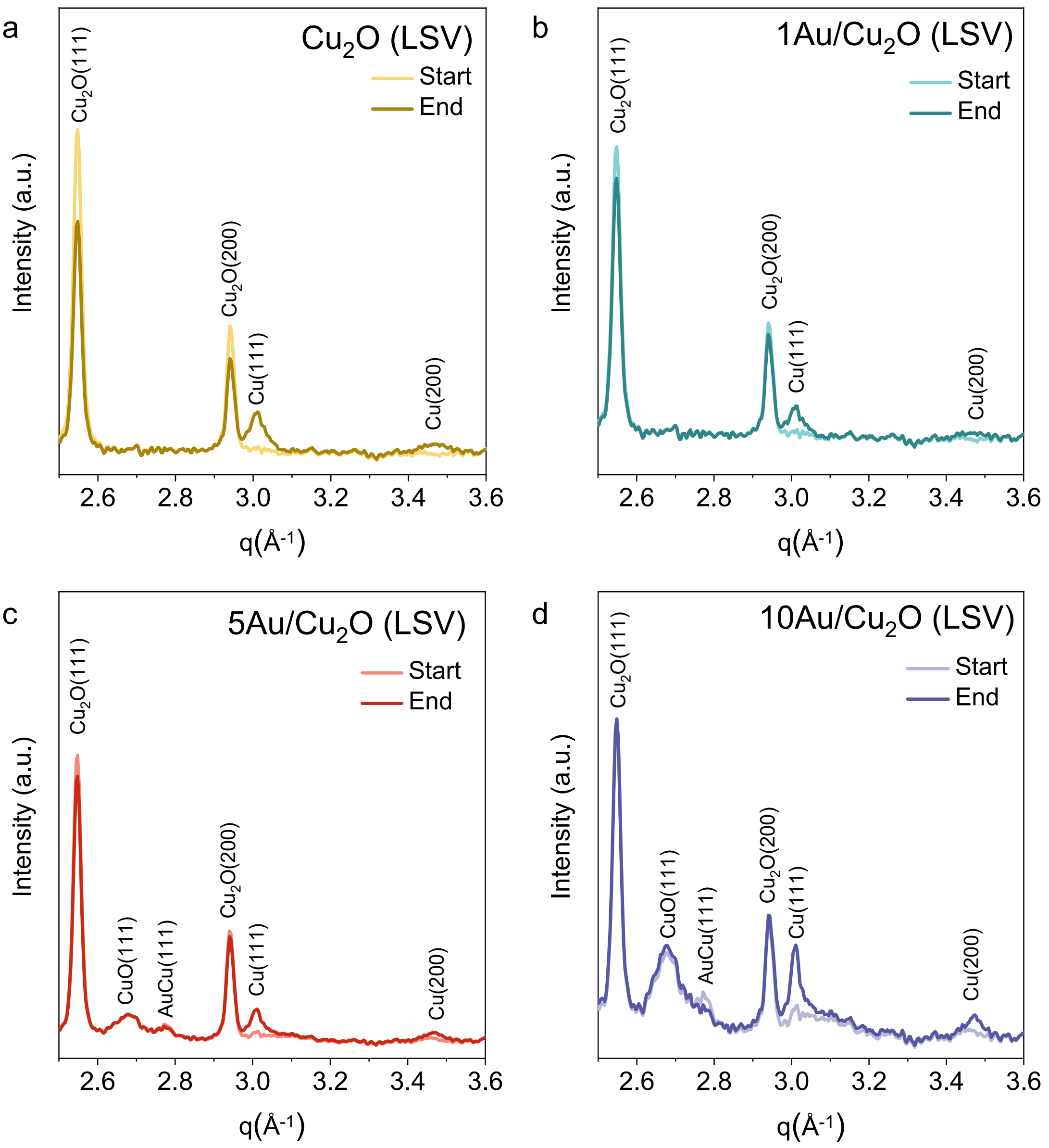

Fig. S8 Overlap of the recorded diffractograms at the start and at the end of the LSV for (a) Cu_2O, (b) 1Au/Cu_2O, (c) 5Au/Cu_2O and (d) 10Au/Cu_2O.

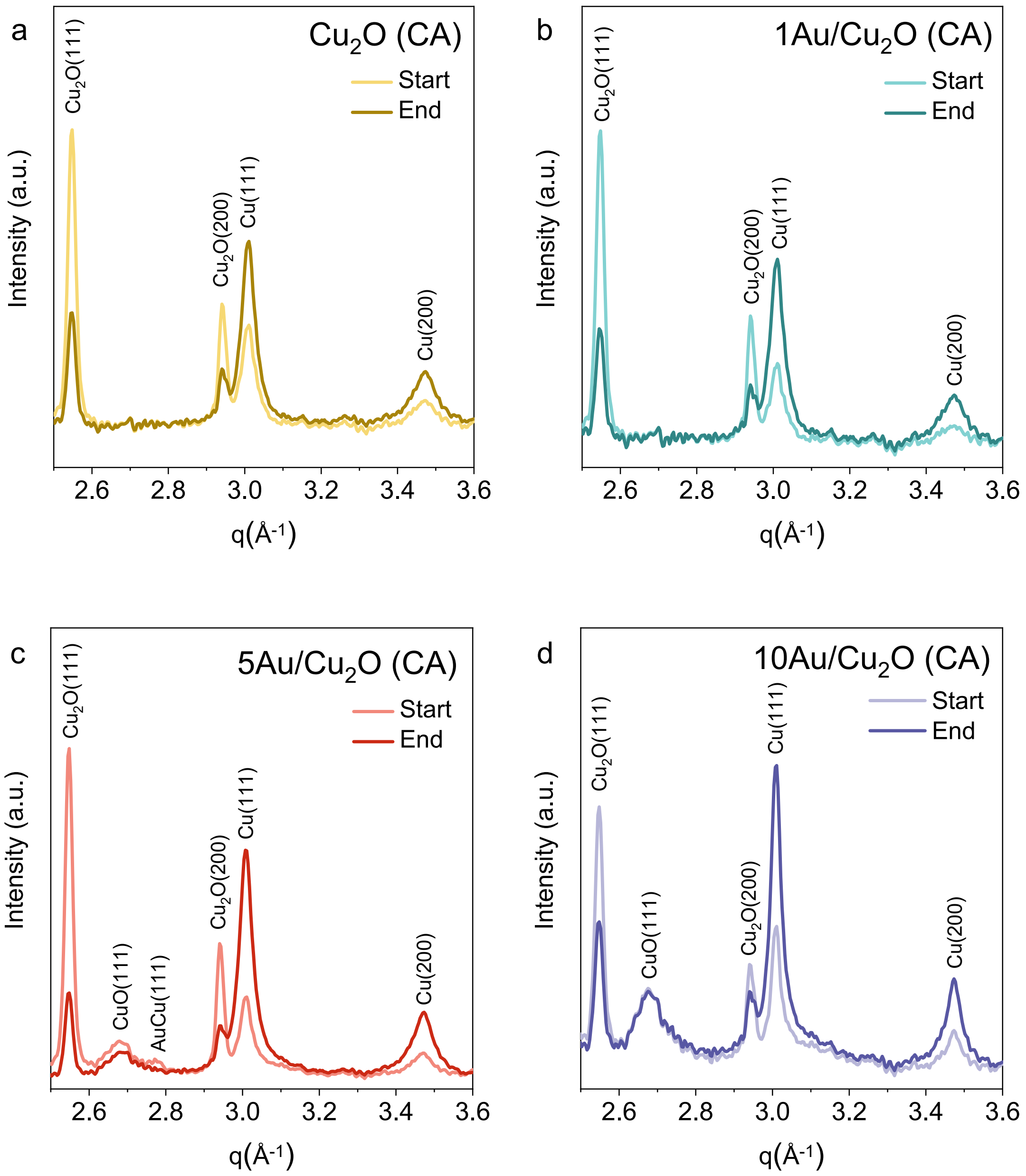

Fig. S9 Overlap of the recorded diffractograms at the start and at the end of the chronoamperometry (CA) for (a) Cu_2O, (b) $1Au/Cu_2O$, (c) $5Au/Cu_2O$ and (d) $10Au/Cu_2O$.

Table S4 Crystallite sizes as determined by the Scherrer equation for the in situ XRD measurements.

Sample	Measurement	Cu_2O (111) crystallite size (nm)	Cu_2O (200) crystallite size (nm)	Cu (111) crystallite size (nm)	Cu (200) crystallite size (nm)
Cu_2O	Fresh	20.3	21.4	-	-
	CV (end)	20.9	21.5	-	-
	LSV (end)	20.5	21.1	12.1	5.2
	CA (end)	20.8	22.7	11.6	7.1
$1Au/Cu_2O$	Fresh	20.0	20.9	-	-
	CV (end)	20.2	21.2	-	-
	LSV (end)	20.8	20.9	14.2	4.5
	CA (end)	19.8	22.3	10.9	6.4
$5Au/Cu_2O$	Fresh	22.8	21.7	-	-
	CV (end)	22.9	21.9		
	LSV (end)	23.1	26.5	8.2	7.8
	CA (end)	23.1	35.7	10.9	8.4
$10Au/Cu_2O$	Fresh	22.3	26.4	-	-
	CV (end)	22.0	23.2	-	-
	LSV (end)	20.9	27.3	8.5	7.6
	CA (end)	21.5	30.1	11.6	9.2

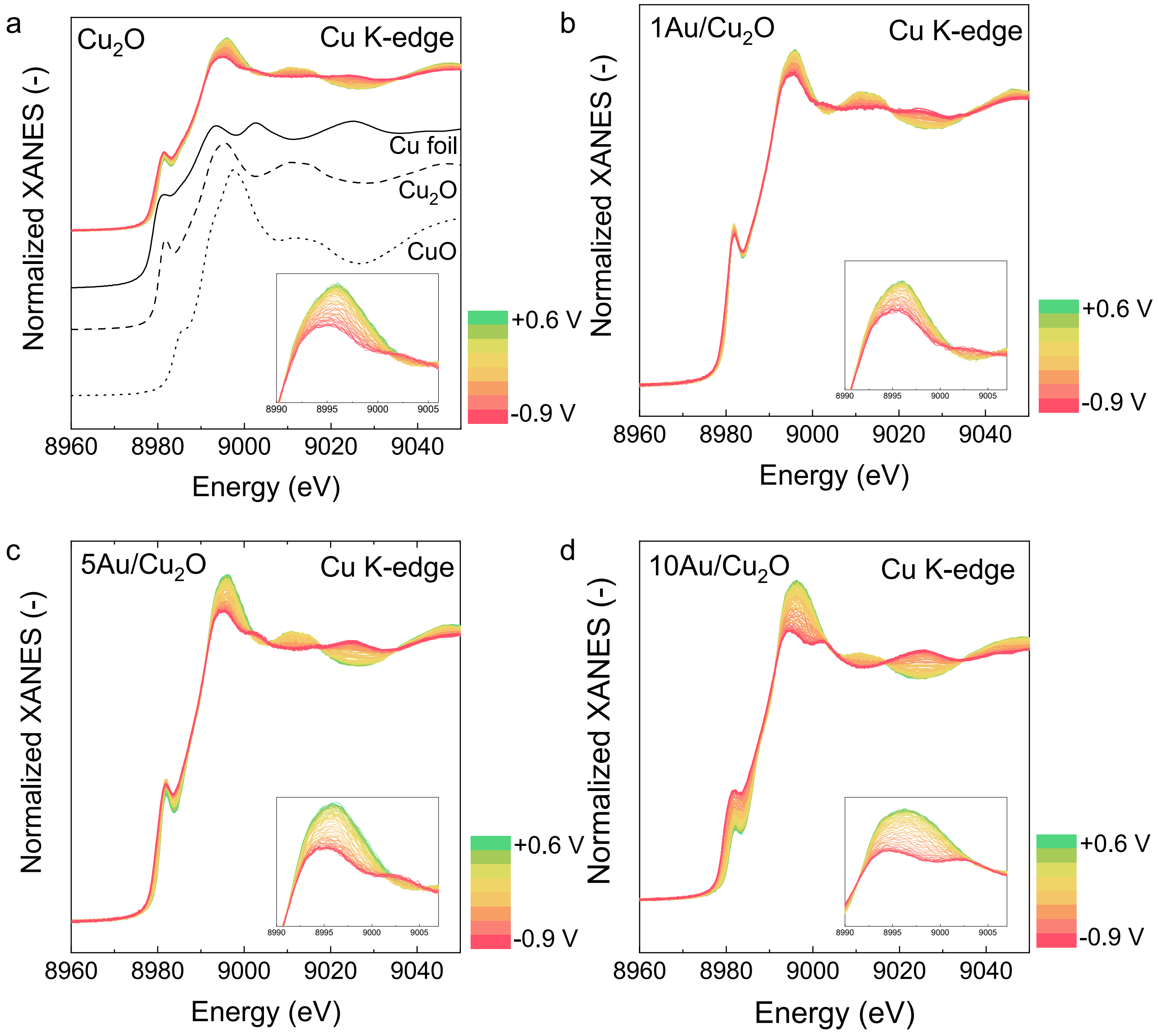

Fig. S10 Evolution of the Cu K-edge XANES spectra during the LSV from +0.6 V to -0.9 V vs. RHE (scan rate: 5 mV/s) for (a) Cu_2O, (b) 1Au/Cu_2O, (c) 5Au/Cu_2O and (d) 10Au/Cu_2O.

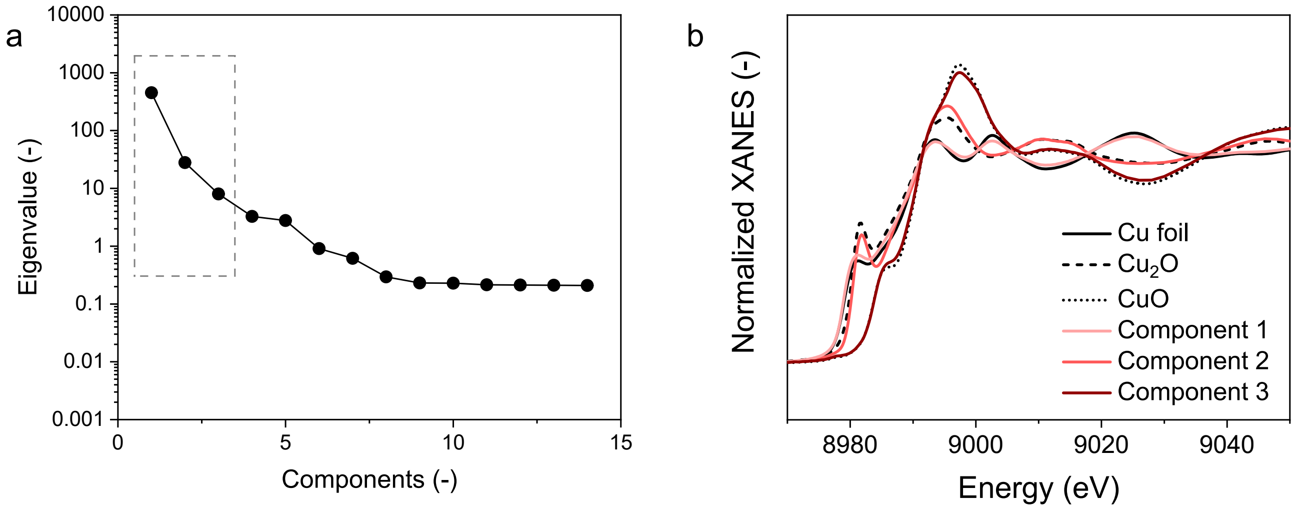

Fig. S11 (a) Scree plot of the in-situ XANES dataset obtained through principal component analysis (PCA). (b) XANES spectra of the three components together with the references.

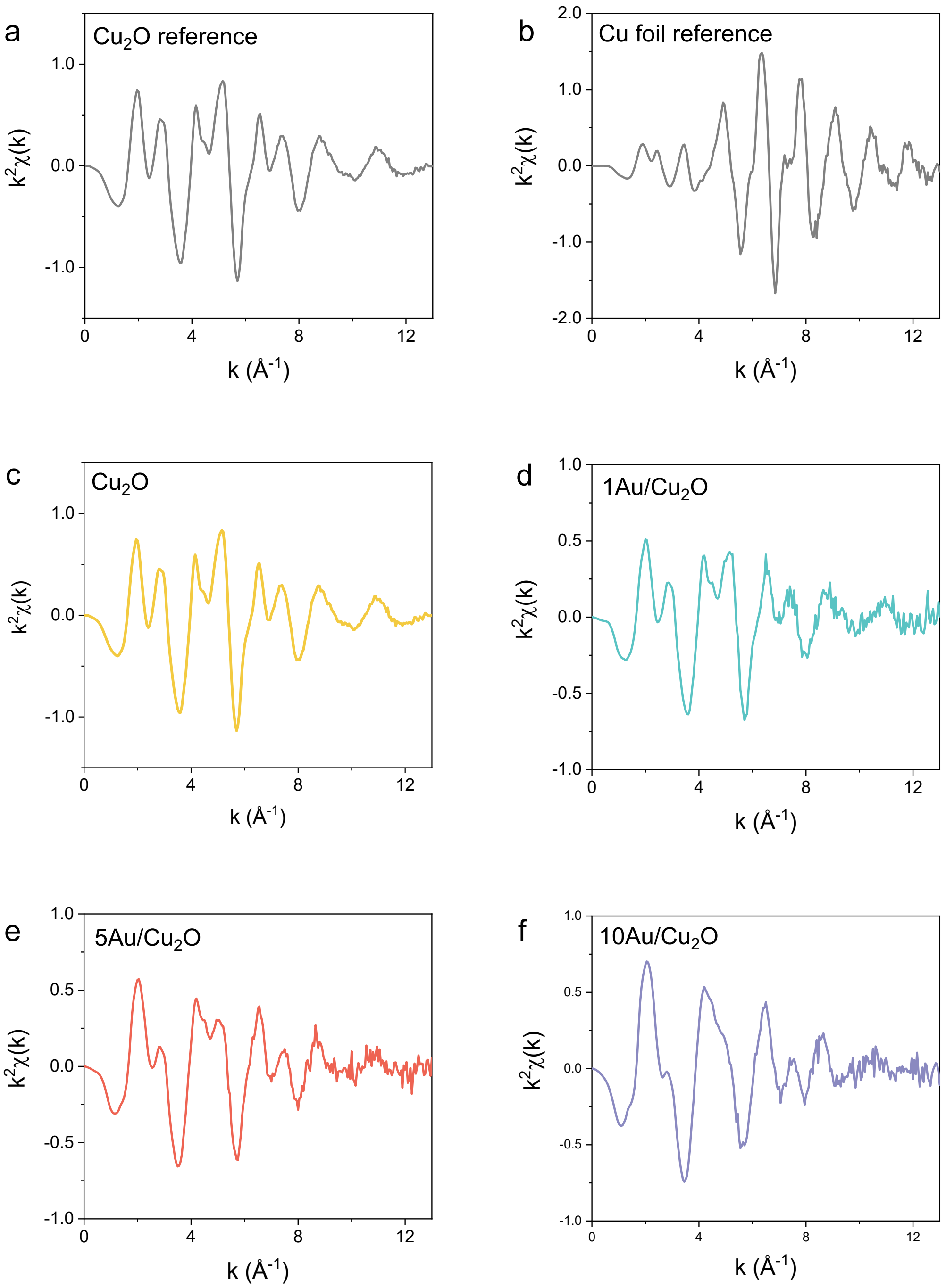

Fig. S12 Fourier-filtered Cu K-edge EXAFS spectra in k-space for the references (a) Cu_2O and (b) Cu foil, and the as-prepared samples of (c) Cu_2O, (d) 1Au/Cu_2O, (e) 5Au/Cu_2O and (f) 10Au/Cu_2O.

Table S5 EXAFS fitting parameters. The data is measured in fluorescence mode. An S_0^2 value of 0.81 was calculated from fitting of the Cu_2O reference and was used for the fitting of FT-EXAFS spectra. An S_0^2 value of 0.61 was calculated from fitting of the Cu foil and was used for fitting of the FT-EXAFS spectra of the samples during chronoamperometry (CA). The calculations were carried out using the software Artemis.

Sample	Measurement	Path	R (Å)	CN (-)	σ^2 ($Å^2$)	ΔE_0 (eV)	R-factor (%)
**Cu_2O**	Reference	Cu-O (oxide)	1.855 ± 0.016	2.00 ± 0.19	0.003 ± 0.001	7.6 ± 1.2	0.6
**Cu foil**	Reference	Cu-Cu (metal)	2.548 ± 0.008	12.06 ± 0.57	0.009 ± 0.0004	4.3 ± 0.5	0.1
**Cu_2O**	Fresh	Cu-O (oxide)	1.892 ± 0.053	1.84 ± 0.13	0.007 ± 0.001	8.4 ± 0.9	0.4
	CA at -0.9 V (last spectrum)	Cu-Cu (metal)	2.556 ± 0.0010	8.54 ± 0.61	0.009 ± 0.0006	3.67 ± 0.8	0.2
	CA at -0.9 V (last spectrum)	Cu-O (oxide)	1.8389 ± 0.0009	0.29 ± 0.24	0.004 ± 0.012		
**$1Au/Cu_2O$**	Fresh	Cu-O (oxide)	1.863 ± 0.025	1.41 ± 0.09	0.005 ± 0.001	7.4 ± 0.9	0.3
	CA at -0.9 V (last spectrum)	Cu-Cu (metal)	2.546 ± 0.010	7.44 ± 0.64	0.009 ± 0.0007	3.3 ± 0.9	0.3
	CA at -0.9 V (last spectrum)	Cu-O (oxide)	1.832 ± 0.007	0.40 ± 0.23	0.002 ± 0.007		
**$5Au/Cu_2O$**	Fresh	Cu-O (oxide)	1.884 ± 0.045	1.73 ± 0.09	0.007 ± 0.001	6.7 ± 0.7	0.3
	CA at -0.9 V (last spectrum)	Cu-Cu (metal)	2.545 ± 0.011	8.52 ± 0.59	0.009 ± 0.0006	3.0 ± 0.7	0.2
	CA at -0.9 V (last spectrum)	Cu-O (oxide)	1.850 ± 0.011	0.26 ± 0.22	0.003 ± 0.01		
**$10Au/Cu_2O$**	Fresh	Cu-O (oxide)	1.934 ± 0.095	2.30 ± 0.20	0.009 ± 0.002	8.2 ± 1.1	0.4
	CA at -0.9 V (last spectrum)	Cu-Cu (metal)	2.548 ± 0.012	8.74 ± 0.51	0.009 ± 0.0005	3.1 ± 0.6	0.2
	CA at -0.9 V (last spectrum)	Cu-O (oxide)	1.889 ± 0.050	0.25 ± 0.19	0.003 ± 0.01		

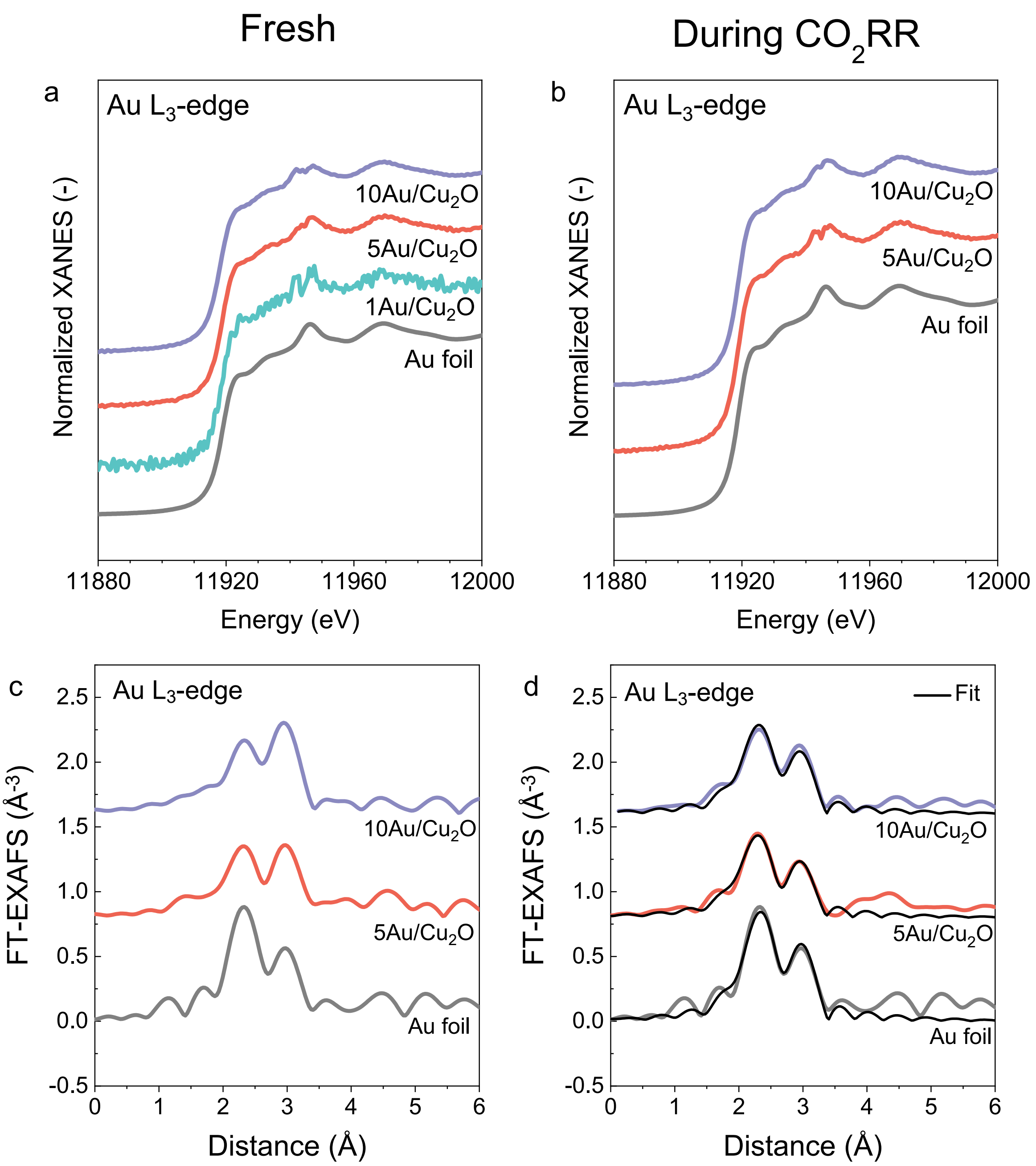

Fig. S13 Normalized Au L_3-edge XANES spectra of the (a) fresh samples and (b) during CO_2RR at -0.9 V vs. RHE. Fourier-transformed Au L_3-edge FT-EXAFS of the (c) fresh samples and (d) during CO_2RR at -0.9 V vs. RHE with corresponding fits (black). Reference (grey) is shown for comparison.

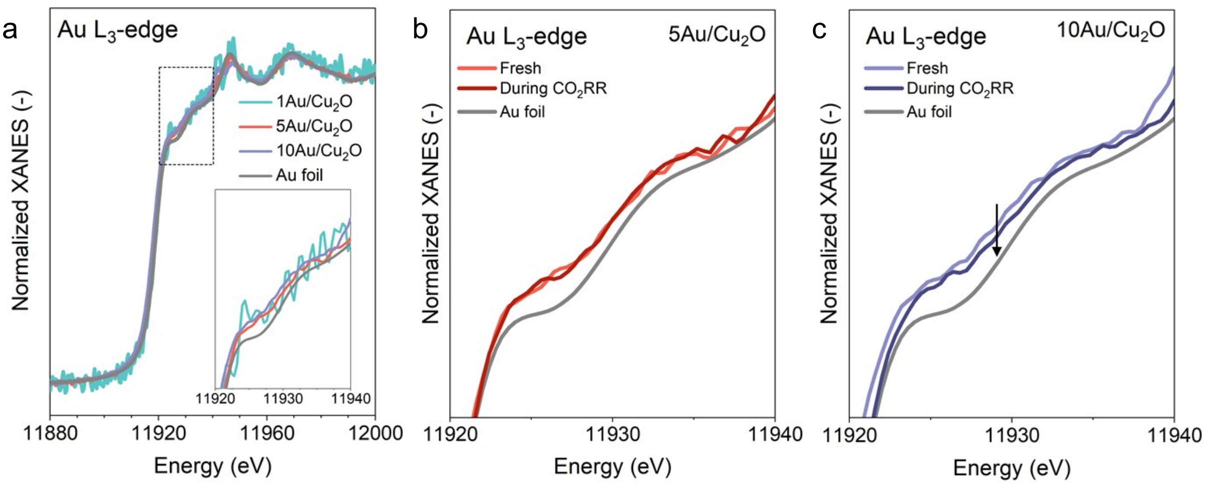

Fig. S14 (a) Overlap of the normalized Au L_3-edge XANES spectra of the fresh samples and the Au foil reference. The increase in the white-line for the Au/Cu_2O samples indicates an electron transfer from Au to Cu. (b,c) Overlap of the normalized Au L_3-edge XANES spectra in the 11920-11940 eV region of the fresh samples and during CO_2RR for 5Au/Cu_2O and 10Au/Cu_2O, respectively. The decreasing white-line intensity, particularly visible for 10Au/Cu_2O, suggests the formation of a more metallic character under reaction conditions.

Table S6 EXAFS fitting parameters. The data is measured in fluorescence mode. An S_0^2 value of 0.87 was calculated from fitting of the Au foil reference and was used for the fitting of FT-EXAFS spectra. The calculations were carried out using the software Artemis.

Sample	Measurement	Path	R (Å)	CN (-)	σ^2 ($Å^2$)	ΔE_0 (eV)	R-factor (%)
**Au foil**	Reference	Au-Au (metal)	2.862 ± 0.022	11.93 ± 1.61	0.008 ± 0.001	0.1 ± 1.0	1.1
**5Au/Cu_2O**	CA at -0.9 V (last spectrum)	Au-Au (metal)	2.838 ± 0.045	10.57 ± 1.67	0.011 ± 0.002	0.88 ± 1.1	1.5
**10Au/Cu_2O**	CA at -0.9 V (last spectrum)	Au-Au (metal)	2.838 ± 0.045	9.74 ± 1.77	0.009 ± 0.002	0.3 ± 1.3	1.7

References

(1) Velasco-Vélez, J-J.; Chuang, C-H.; Gao, D.; Zhu, Q.; Ivanov, D.; Jeon, H. S; Arrigo, R.; Mom, R. V.; Stotz, E.; Wu, H-L.; Jones, T. E.; Roldan Cuenya, B.; Knop-Gericke, A.; Schlögl, R. On the Activity/Selectivity and Phase Stability of Thermally Grown Copper Oxides during the Electrocatalytic Reduction of CO_2. *ACS Catal.* **2020**, *10*, 11510-11518.
